# Ack1 Mediated AKT/PKB Tyrosine 176 Phosphorylation Regulates Its Activation

**DOI:** 10.1371/journal.pone.0009646

**Published:** 2010-03-19

**Authors:** Kiran Mahajan, Domenico Coppola, Sridevi Challa, Bin Fang, Y. Ann Chen, Weiwei Zhu, Alexis S. Lopez, John Koomen, Robert W. Engelman, Charlene Rivera, Rebecca S. Muraoka-Cook, Jin Q. Cheng, Ernst Schönbrunn, Said M. Sebti, H. Shelton Earp, Nupam P. Mahajan

**Affiliations:** 1 Drug Discovery Program, Moffitt Cancer Center, Tampa, Florida, United States of America; 2 Department of Anatomic Pathology, Moffitt Cancer Center, Tampa, Florida, United States of America; 3 Department of Experimental Therapeutics, Moffitt Cancer Center, Tampa, Florida, United States of America; 4 Proteomics Facility, Moffitt Cancer Center, Tampa, Florida, United States of America; 5 Biostatistics Division, Moffitt Cancer Center, Tampa, Florida, United States of America; 6 Department of Molecular Oncology, Moffitt Cancer Center, Tampa, Florida, United States of America; 7 Lineberger Comprehensive Cancer Center, Department of Pharmacology, University of North Carolina, Chapel Hill, North Carolina, United States of America; Institute of Cancer Research, United Kingdom

## Abstract

The AKT/PKB kinase is a key signaling component of one of the most frequently activated pathways in cancer and is a major target of cancer drug development. Most studies have focused on its activation by Receptor Tyrosine Kinase (RTK) mediated Phosphatidylinositol-3-OH kinase (PI3K) activation or loss of Phosphatase and Tensin homolog (PTEN). We have uncovered that growth factors binding to RTKs lead to activation of a non-receptor tyrosine kinase, Ack1 (also known as ACK or TNK2), which directly phosphorylates AKT at an evolutionarily conserved tyrosine 176 in the kinase domain. Tyr176-phosphorylated AKT localizes to the plasma membrane and promotes Thr308/Ser473-phosphorylation leading to AKT activation. Mice expressing activated Ack1 specifically in the prostate exhibit AKT Tyr176-phosphorylation and develop murine prostatic intraepithelial neoplasia (mPINs). Further, expression levels of Tyr176-phosphorylated-AKT and Tyr284-phosphorylated-Ack1 were positively correlated with the severity of disease progression, and inversely correlated with the survival of breast cancer patients. Thus, RTK/Ack1/AKT pathway provides a novel target for drug discovery.

## Introduction

Protein kinase AKT plays a central role in growth, proliferation and cell survival [1], [2], [3]. AKT activation occurs when ligand binding to RTK facilitates translocation of AKT to the plasma membrane [4], [5], [6], [7] where it is phosphorylated at Thr308 by phosphoinositide-dependent protein kinase-1 (PDK1) and at Ser473 by the ‘PDK2’, a class of about 10 different kinases [8] including the mTORC2 complex [9]. Phosphorylation of AKT at Thr308 and Ser473 leads to its kinase activation [10]. Upon activation, AKT phosphorylates its substrates to transduce survival signals [1], [3], [11], [12]. During AKT activation, the first step is the production of phosphatidylinositol 3,4,5 trisphosphate (PIP3) by PI3K. PDK1 and AKT bind the phospholipid PIP3 via their PH domains and are recruited to the plasma membrane. While RTK/PI3K mediated recruitment of AKT to the plasma membrane is a well characterized mechanism, mounting evidence indicate that AKT activation can occur in a PI3K-independent fashion [13], [14], [15], [16], [17], [18]. About a third of the breast and prostate tumors and majority of the pancreatic tumors that exhibit AKT activation, retain normal PTEN and PI3K activity [15] [19], [20]. Interestingly, normal PTEN expression was also seen in breast, ovarian and prostate tumors that exhibit activated AKT [15]. While RTKs are suggested to be involved [21], the molecular mechanisms regulating RTK mediated AKT activation in cancers with normal PTEN and PI3K activity is poorly understood [22]. Further, *PIK3CA* activating mutation has recently been shown to be neither necessary nor sufficient for full AKT activation in situ [23]. Thus, collectively these data suggest the existence of additional pathways that regulate AKT activation in response to growth factors.

Ack1, a nonreceptor tyrosine kinase has emerged as a critical early transducer of variety of extracellular growth factor stimuli including heregulin, insulin, EGF and PDGF signaling [24], [25], [26], [27], [28]. Ack1 is ubiquitously expressed and primarily phosphorylated at Tyr284 leading to its kinase activation [25], [27]. Our earlier studies demonstrated that Ack1 regulates prostate cancer progression to androgen independence by positively regulating androgen receptor (AR) and negatively regulating the tumor suppressor, Wwox [25], [26], [29]. Ack1 gene is also shown to be amplified in primary lung, ovarian and prostate tumors which correlated with poor prognosis [30]. In this report, we have identified a novel mechanism of Ack1 mediated AKT activation wherein phosphorylation of Tyrosine 176 in the AKT kinase domain results in its translocation to the plasma membrane and subsequent kinase activation.

## Results

### Ack1 Phosphorylates AKT at Evolutionary Conserved Tyr176 Resulting in AKT Activation

**Figure 1:**
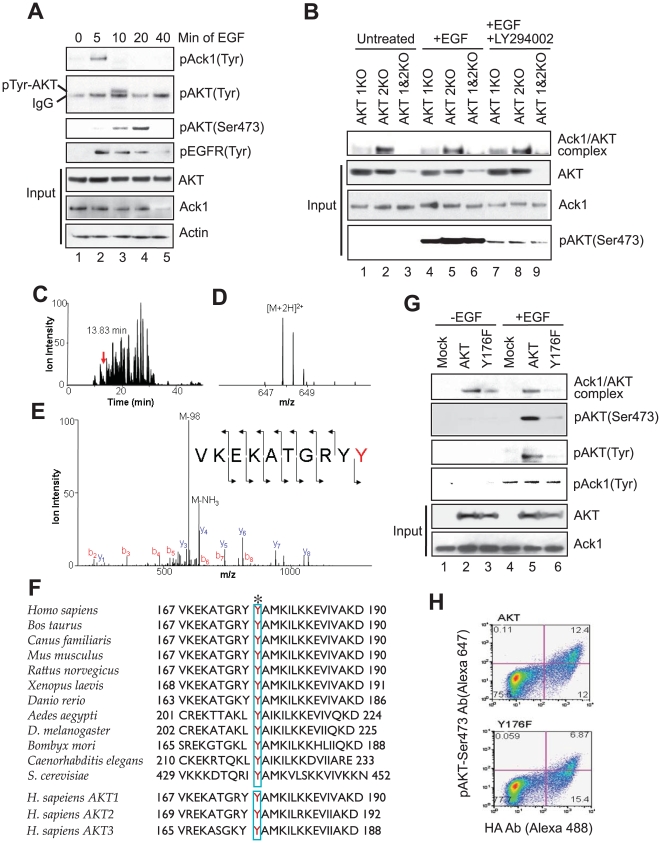
Tyr176 phosphorylation precedes AKT activation. (**A**) MEF2KO cells were serum starved (24 h) and treated with EGF (10 ng/ml). The lysates were immunoprecipitated or IP with anti-Ack1 (top panel), anti-AKT (second panel) and anti-EGFR (fourth panel) antibodies followed by immunoblotting or IB with anti-pTyr antibodies. Remaining panel represents IB with antibodies as shown. (**B**) MEFs were serum starved (24 h) and treated with EGF (10 ng/ml for 10 mins) or pretreated with LY294002 (10 µM for 1 h) and EGF. The lysates were IP with Ack1 antibodies followed by IB with pan-AKT antibodies (top panel). (**C**) HA-tagged Tyr-phosphorylated AKT was purified (see Fig. S2A) followed by trypsin/chymotrypsin digestion. The peptide was detected at 13.83 mins in the total ion chromatogram (**C**) with mass-to-charge ratio 647.8132, which represents an error of 0.38 ppm (**D**)**.** (**E**) The tandem mass spectrum matched the sequence, VKEKATGRY**pY** indicating that the C-terminal tyrosine was phosphorylated; the detection of the phosphotyrosine y_1_ is consistent with this localization. (**F**) Alignment of AKT protein sequences revealed that tyrosine at 176 is invariant from yeast to humans and all the three known human AKT isoforms. (**G**) MEF1&2KO cells expressing HA-tagged AKT or Y176F mutant were serum-starved (24 h), treated with EGF for 15 mins and lysates were IP with anti-Ack1 Abs followed by IB with anti-AKT antibodies (top panel). The lysates were also IP with anti-Ack1 antibodies followed by IB with pTyr antibodies (panel 4). The same blot was stripped and IB with anti-Ack1 antibodies (Bottom panel). These lysates were also subjected to IP with anti-HA antibodies followed by IB with Ser473, pTyr and AKT antibodies (panels 2, 3 and 5, respectively). (**H**) Flow cytometry of AKT and Y176F mutant expressing MEF1&2KO cells. Cells were serum starved for 24 h, treated with EGF for 15 mins, fixed and stained with HA-antibodies conjugated to Alexa488 and phosphoSer473-antibodies conjugated to Alexa 647. Upper right quadrant represents cells which express HA-tagged AKT or Y176F mutant that are also Ser473-phosphorylated.

We observed that EGF treatment of mouse embryonic fibroblasts (MEFs) resulted in rapid Tyr-phosphorylation of Ack1 as well as Akt1 at 5 and 10 mins respectively, suggesting that these two Tyr-phosphorylation events could be linked (**Fig. 1A**). To test this hypothesis, we examined whether Ack1 could bind and Tyr-phosphorylate AKT following RTK activation. Co-immunoprecipitation of lysates derived from Akt1, Akt2, and Akt1& 2 knockout mouse embryo fibroblasts (MEF1KO, MEF2KO, and MEF1&2KO, respectively, **Fig. S1A**) that were treated with EGF, either with or without pretreatment with LY294002, a PI3K inhibitor, revealed that endogenous Akt1 (AKT here onwards) and Ack1 formed a stable complex which was not abrogated by LY294002 (**Fig. 1B**). The bottom panel shows that upon LY294002 addition there was substantial decrease in AKT Ser473-phosphorylation, suggesting that LY294002 is functional. Akt2 interacted weakly with Ack1, while Akt3 present at low levels in the MEF1&2KO cells was not detectable in the complex.

To test whether Ack1 directly phosphorylates AKT, *in vitro* binding assay was performed and AKT Tyr-phosphorylation was assessed. Myc-tagged Ack1 and HA-tagged AKT constructs were expressed and purified using respective antibody beads followed by elution, as described in methods section (**Fig. S1B**). *In vitro* binding assay revealed that purified Ack1 interacted directly with AKT resulting in AKT Tyr176-phosphorylation (**Fig. S1B–D**). Further, we generated GST-Ack construct that harbors kinase, SH3 and CRIB domain (schematic shown in **Fig. S1E**) and expressed it in *E. coli* (**Fig. S1E**) [25], [31]. Androgen-receptor (AR), another Ack1 substrate [26] was expressed as FLAG-tagged construct in HEK293 cells and purified using FLAG-beads (**Fig. S1E**, left panel). GST-tagged Ack1 or GST (as control) bound to glutathione beads were incubated with purified AKT or Y176F mutant of AKT or AR (shown in **Fig. S1B** and **E**). GST-Ack1 bound to purified AKT and AR but not the Y176F mutant of AKT suggesting that AKT and AR are direct binding partners of Ack1 (**Fig. S1F**).

Affinity purification of AKT coexpressed with Ack1 (**Fig. S2A**), followed by mass spectrometry analysis revealed that AKT was phosphorylated at Tyrosine 176 (**Fig. 1C–E**). Tyr176, located in the kinase domain, is evolutionarily conserved from unicellular eukaryotes to mammals and within all the three AKT isoforms (**Fig. 1F**). Two other phosphorylation events, Ser473 and Thr308 were also identified in the same preparation (**Fig. S2B–G**). *In-silico* analysis revealed that Tyr176 and Ser473 are located in regions with increased conformational flexibility and phosphorylation at Tyr176 is likely to induce substantial conformational change and thus affect the loop harboring Ser473 (**Fig. S3**). To determine whether AKT Tyr176-phosphorylation is an upstream event that regulates AKT activation (or Ser473 phosphorylation, hereafter), site directed mutagenesis was performed to generate AKT phospho-tyrosine (Y176F) mutant (**Fig. S4A**). The Y176F mutant interacted poorly with Ack1 in the absence of ligand, and in the presence of ligand failed to interact with Ack1 resulting in decreased AKT Tyr/Ser-phosphorylations (**Fig. 1G**, lane 6). Flow cytometric analysis of EGF treated cells revealed significant reduction in Ser473-phosphorylation in MEF1&2KO cells expressing Y176F as compared to AKT (**Fig. 1H** and **Fig. S4B**). These results imply that Ack1 mediated AKT Tyr-phosphorylation results in subsequent AKT activation.

### Ack1/AKT Interacting Domains

To identify domains involved in Ack1-AKT interaction, various deletions of Ack1 and AKT were generated (**Fig. S4A**). MEF1&2KO cells were co-transfected with HA-tagged AKT deletions and activated Ack1 or caAck. Immunoprecipitation using HA antibodies followed by immunoblotting with pTyr antibodies revealed Tyr-phosphorylation of full-length AKT and AKT lacking carboxy terminus (ΔCT-AKT), however, AKT deletion construct lacking the PH domain (ΔPH-AKT) exhibited significant decrease in Tyr-phosphorylation (**Fig. S4C**, top panel). The decreased phosphorylation of AKT deletion construct lacking PH domain could be due to poor binding with activated Ack1. To assess this interaction in further detail, co-immunoprecipitation experiment was performed. It revealed that in contrast to AKT or ΔCT-AKT, ΔPH-AKT weakly binds Ack1 (**Fig. S4D**, top panel). We have demonstrated that Tyr176 residue in AKT kinase domain is necessary for Ack1/AKT interaction, thus, collectively it indicates that the Ack1 need both the PH domain and tyrosine176 in AKT kinase domain for complex formation.

To identify the region in Ack1 that recognize AKT, MEF1&2KO cells were transfected with Myc-tagged Ack1 deletions (shown in **Fig. S4A**) and HA-tagged AKT. The lysates were immunoprecipitated using Myc antibodies followed by immunoblotting with AKT antibodies. The Ack1 construct expressing SAM and kinase domains (cAck) was able to bind AKT, however, construct lacking a part of kinase domain (dAck) bound poorly to endogeneous AKT (**Fig. S4E**, top panel). GST-Ack1 that possess Kinase-SH3-CRIB domains but lacking SAM domain was able to bind AKT (**Fig. S1F**). Taken together it indicates that the kinase domain in Ack1 and tyrosine176 in the kinase domain along with AKT PH domain appear to be minimal domains required for efficient Ack1/AKT complex formation.

### Somatic Autoactivating Mutation (E346K) in Ack1 Activates AKT

While growth factor binding to RTK or amplification of the Ack1 gene causes Ack1 kinase activation [25], [26], [30], somatic autoactivating mutations in Ack1 have not yet been identified. Recently, four point mutations in Ack1, i.e. R34L, R99Q, E346K, M409I have been identified in the COSMIC database. Using site-directed mutagenesis, we generated HA-tagged point mutants (**Fig. S5A**). We tested these mutants and observed that E346K mutant undergoes autoactivation and causes AKT Tyr/Ser/Thr-phosphorylation in serum starved cells (**Fig. S5B** and **C**). Earlier we and others have characterized a point mutant (L487F mutation) that leads to constitutive activation of Ack1, also called caAck [26], [32]. Both caAck(L487F mutant) and E346K autoactivating mutant of Ack1 exhibited Tyr284-phosphorylation in the activation loop (**Fig. S5D**). We also measured the intrinsic kinase activity of the Y176F mutant and the wildtype AKT in the absence and presence of activated Ack1. The wildtype AKT displays significant increase in the kinase activity as compared to the Y176F mutant when coexpressed with either one of the Ack1 constructs, E346K and caAck (**Fig. S5E** and **F**). These results demonstrate that the somatic autoactivating mutations in Ack1 are sufficient to activate AKT. Taken together with the earlier evidence indicating direct Ack1-AKT interaction, it opens an intriguing possibility of RTK/PI3K-independent AKT activation in tumors that is mediated by (auto) activated Ack1.

### Tyr176-Phosphorylated AKT Translocates to the Plasma Membrane Leading to AKT Activation

**Figure 2:**
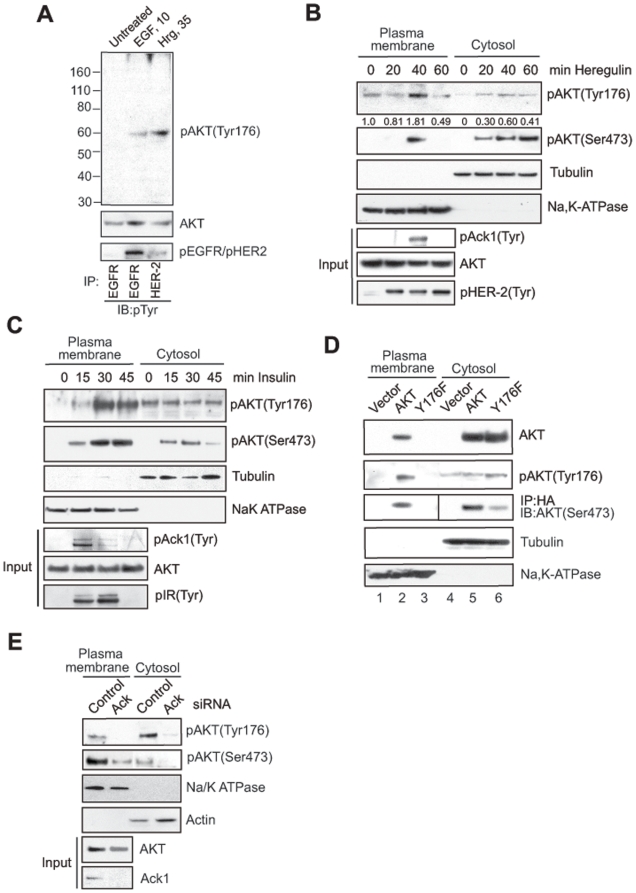
Tyr176-phosphorylation regulates AKT plasma membrane localization. (**A**) RWPE, normal prostate epithelial cells were treated with EGF (10 ng/ml,10 mins) and heregulin (10 ng/ml, 35 mins), whole cell protein lysates were subjected to IB with indicated antibodies. (**B, C**) MCF-7 cells were serum starved (24 h) and treated with (**B**) insulin (50 ng/ml) or (**C**) heregulin (30 ng/ml) for indicated times. Cell lysates were fractionated and IB with the indicated antibodies. Input panels pAck1(Tyr), pIR(Tyr) and pHER-2(Tyr) represents IP with respective antibodies followed by IB with pTyr antibodies. (**D**) MEF 1&2KO cells were transfected with HA-tagged AKT or Y176F mutant, serum starved (24 h) and treated with EGF for 15 mins. Cell lysates were fractionated and IB with anti-HA (top panel) and indicated antibodies (bottom panels). (**E**) MCF7 cells were transfected with control or Ack1-specific siRNAs (40 nM) for 48 h and treated with heregulin for 40 mins. Cell lysates were fractionated and IB with indicated antibodies. In this experiment we have used half the volumes buffer for extraction of cytosolic proteins. Thus, the cytosolic extracts are 2X concentrated as compared to Fig. 2B–C, which explains more p176-AKT in cytosol fraction than the plasma membrane fraction.

Mechanistically, targeting AKT to the plasma membrane is necessary for AKT activation [1], [6], [7], [13]. Loss of the PH domain resulted in decrease in AKT Tyr-phosphorylation upon co-expression with activated Ack1 (**Fig. S4A, C** and **D**). Further, Ack1 interacts with RTKs which are located in the membrane [25], [26], [28]. These attributes suggest that activated Ack1 could engage AKT at the plasma membrane. To investigate the role of AKT Tyr176-phosphorylation on its cellular compartmentalization, we generated phospho-antibodies that specifically recognized Tyr176-phosphorylated AKT or pTyr176-AKT (details in SI methods). The antibodies were extensively validated (**Fig. 2A**, **Fig. S6A**, also see top panels of **Fig. 2B, C and E**, **Fig. S6B**). Normal prostate epithelial cells, RWPE, exhibited pTyr176-AKT expression upon treatment with EGF and heregulin ligand (**Fig. 2A**). The pTyr176-AKT was detected when activated Ack1 was coexpressed with AKT but not the Y176F mutant. Further, incubation of the pTyr176-AKT-antibody with phosphoAKT-Y176-peptide resulted in loss of binding to Tyr176-phosphorylated AKT (**Fig. S6A**). Cell fractionation studies revealed that heregulin, insulin and EGF treatment resulted in a time-dependent accumulation of pTyr176-AKT at the plasma membrane that lead to AKT activation (**Fig. 2B, C** and **Fig. S6B**, top panels). Optimal AKT Tyr-176 phosphorylation and plasma membrane accumulation was observed at 10, 30 and 40 mins upon EGF, insulin and heregulin ligand treatments, respectively (**Fig. S6B** and **Fig. 2B, C**). To assess whether EGF mediated AKT activation is dependent upon Tyr176-phosphorylation, MEF1&2KO cells expressing AKT or Y176F mutant were treated with EGF ligand. The Y176F mutant failed to translocate to the plasma membrane and become activated by EGF (**Fig. 2D**). The basal levels of pTyr176-AKT seen in cytosolic fraction (**Fig. 2D**, panel 2, lanes 4–6) is likely to be Tyr-phosphorylated AKT3. Depletion of Ack1 by siRNA abrogated heregulin mediated AKT Tyr176-phosphorylation, plasma membrane localization and activation in MCF-7 cells (**Fig. 2E**) and MEFs (unpublished data). Further, GFP-E346K recruited dsRed-AKT but not the dsRed-Y176F mutant to the plasma membrane as assessed by immunofluorescence (**Fig. S6C–J**). Taken together, these data suggest that Ack1 is a key intermediate signaling entity necessary for RTK mediated AKT Tyr176-phosphorylation.

### Ack1 Facilitates AKT Plasma Membrane Localization and Activation

**Figure 3:**
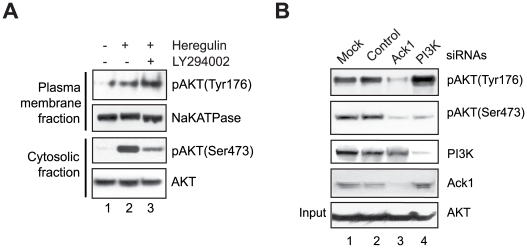
Tyr176-phosphorylation of AKT is PI3K-independent. (**A**) MCF-7 cells were pretreated with LY294002 (10 µM, 1 h) followed by heregulin for 40 mins. Cell lysates were fractionated and membrane fraction was subjected to IB with indicated antibodies. (**B**) MCF-7 cells were mock transfected or transfected with control, Ack1 and PI3K siRNAs, followed by insulin treatment for 30 mins. Cell lysates were subjected to IP with pTyr-antibodies, followed by IB with pTyr176-AKT antibodies (top panel). Lower panels show IB with indicated antibodies. The experiment was performed with two different Ack1 siRNAs (Qiagen).

Because Ack1/AKT interaction was unaffected by LY294002 treatment (**Fig. 1B**) we assessed whether AKT Tyr176-phosphorylation could occur upon inhibition of PI3K activity. First, LY294002 treatment neither affected endogenous AKT Tyr176-phosphorylation nor its membrane localization (**Fig. 3A**). Second, in contrast to Ack1 knockdown, depletion of PI3K 110α subunit by siRNA did not inhibit pTyr176-AKT levels in MCF7 cells treated with insulin (**Fig. 3B**). However, Ser473 phosphorylation of AKT was reduced upon knockdown of either Ack1 or PI3K, suggesting existence of two distinct pathways of AKT activation. Third, membrane fraction of AKT was phosphorylated at Ser473 even in the presence of LY294002 when coexpressed with activated Ack1 in serum starved MEF1&2KO cells (**Fig. S7A**, panel 2). To determine whether Tyr-phosphorylated AKT can translocate to the plasma membrane in the absence of PIP3, AKT point mutant R25C that binds PIP3 inefficiently [4] was generated (**Fig. S7B**). The R25C mutant was Tyr-phosphorylated and recruited to membrane when coexpressed with activated Ack1, in the absence of ligand (**Fig. S7C** and **D**). Interestingly, in contrast to AKT which bound PIP3, Tyr-phosphorylated AKT bound another membrane phospholipid, phosphatidic acid (PA) (**Fig. S8**). Combined together, our data indicates that RTK/Ack1 pathway could directly facilitate AKT plasma membrane localization and activation and a fraction of AKT that is Tyr176-phosphorylated can translocate to the membrane and undergo Ser473-phosphorylation even when PI3K is inhibited.

### AKT Tyr176-Phosphorylation Suppresses Expression of Apoptotic Genes and Promotes Mitotic Progression

**Figure 4:**
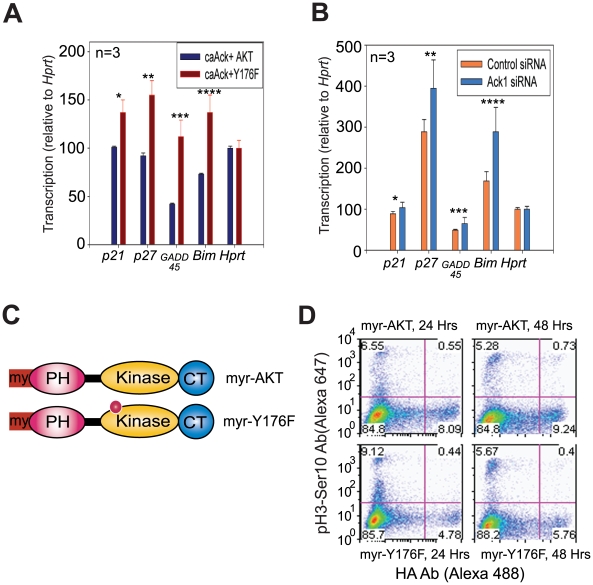
Tyr176 phosphorylated AKT suppresses FoxO gene transcription and promotes cell cycle progression. (**A**) MEF1&2KO cells were transfected with caAck and HA-tagged AKT or Y176F, serum starved (24 h) and harvested. Total RNA was prepared and quantitative RT-PCR was performed. Data are representative of three independent experiments. *p≤0.05; **p≤0.03; ***p≤0.02; ****p≤0.02. (**B**) MEF2KO cells were transfected with control or Ack1-specific siRNAs (40 nM) for 48 h and treated with EGF for 30 mins. Total RNA was prepared and quantitative RT-PCR was performed. *p≤0.01; **p≤0.05; ***p≤0.06; ****p≤0.05. (**C**) Schematic representation of myr-AKT and myr-Y176F point mutants. SDM of myr-AKT was performed to generate the Y176F mutation. PH, Pleckstrin homology domain; Kinase, Kinase domain and CT, Carboxy Terminal regulatory region. (**D**) AKT MEF1&2 KO cells were transfected with HA-tagged myr-AKT or myr-Y176F mutant and harvested 24 h and 48 h post-transfection. Cells were fixed and stained with anti-HA antibodies conjugated with Alexa 488 and anti-pSerine10-Histone3 conjugated with Alexa 647, a marker used to distinguish cells in late G2 and early M phase, and analyzed by flow cytometry. HA-myrAKT expressing cells showed 75% increase in the number of cells undergoing mitosis (upper right quadrant), while, HA-myrY176F-AKT expressing mitotic cells remain unchanged.

Earlier we have observed that Ack1 translocates to the nucleus upon it's Tyr-phosphorylation [26]. We assessed the localization of pTyr176-AKT when Ack1 was activated. Ligand treatment facilitated nuclear translocation of both endogenous pTyr284-Ack1 and pTyr176-AKT (**Fig. S9A**). FoxO subgroup of transcription factors are phosphorylated by AKT leading to rapid relocalization of FoxO proteins from nucleus to cytoplasm, thus, preventing transactivation of target genes [1], [11], [12]. FoxO proteins regulate genes involved in cell cycle arrest (*e.g. p21*, *p27KIP1*), cell death (*e.g*. *Bim-1*) and DNA repair *(e.g. GADD45*) [11]. Real time quantitative RT-PCR analysis revealed that in MEF1&2KO cells co-expressing caAck and AKT, expression of *p21, p27, Bim-1* and *GADD45* is down regulated as opposed to the activated Ack and Y176F mutant co-expressing cells (**Fig. 4A**). Consistent with this observation, depletion of Ack1 protein by siRNA resulted in increased FoxO-responsive gene expression (**Fig. 4B**).

To further understand the molecular role of Tyr176 in cell growth, we generated a HA-tagged myristoylated Y176F or myr-Y176F (**Fig. 4C**). As the myristoylated version of AKT is constitutively anchored at the membrane, it exhibits high levels of AKT activation, as seen by Thr308-phosphorylation (**Fig. S9B**). MEF1&2KO cells expressing myr-Y176F exhibited significant decrease in Thr308-phosphorylation confirming that AKT Tyr176-phosphorylation is an important event for subsequent AKT activation. Further, MEF1&2KO cells expressing myr-AKT grow exponentially as observed by an increase in the number of the double-positive HA and phospho-H3 (Ser10) stained cells, indicative of cells undergoing mitosis (**Fig. 4D**). In contrast, the number of double-positive myr-Y176F expressing cells remained unchanged after 24 hours (**Fig. 4D**). Thus, AKT Tyr176-phosphorylation can both suppress pro-apoptotic gene transcription and promote mitotic progression.

### Probasin-Ack1 Transgenic Mice Display AKT Activation and Develop Prostatic Intraepithelial Neoplasia

**Figure 5:**
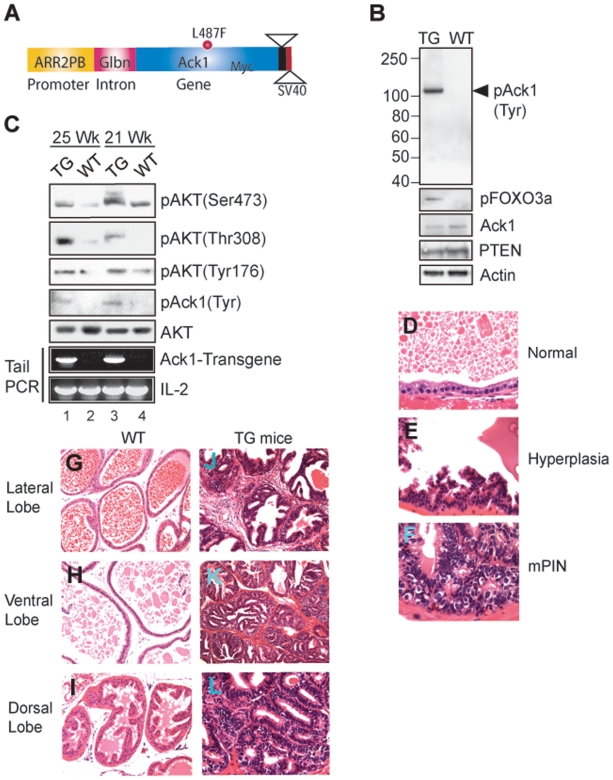
Probasin-Ack1 transgenic mice display pTyr176-AKT and develop mPINs. (**A**) Transgenic construct (Prob-Ack1) is shown. (**B**) A 25 wk old Probasin-Ack1 transgenic (TG) and wild type [21] male mice prostate lysates were subjected to IP using anti-Myc antibodies followed by IB with pTyr antibodies (top panel). For bottom panels, lysates were subjected to IB with indicated antibodies. (**C**) Prostate lysates from 21 and 25 wk old TG and the WT siblings were IB with respective antibodies. The bottom 2 panels represent tail-PCR of these mice. IL-2 was an internal control for PCR. (**D–L**) Haematoxylin and eosin (H&E) stained WT and TG mice prostates. Histological appearance of the prostate lateral lobe from a 22 wk old WT mouse (**D**), and corresponding lobe from age-matched TG mice with intraepithelial hyperplasia (*E*). The lateral prostate from 49 wk old TG mice exhibiting mPIN (**F**) is shown. Contrasting histological appearance of the lateral, ventral and dorsal lobes of the prostate glands from a WT mouse (**G–I**), and corresponding lobes from TG mice (49 week old) are shown (**J–L**).

We generated a transgenic mouse model in which Myc-tagged activated Ack1 was expressed under the control of modified Probasin (PB) promoter, ARR2PB (**Fig. 5A** and **B**). PB-Ack1 transgenic mice (TG) display significant increase in AKT Tyr176-phosphorylation leading to Ser473/Thr308-phosphorylation (**Fig. 5C**, top 3 panels) and AKT substrate FOXO3a Ser318/321-phosphorylation (**Fig. 5B**, panel 2) in the prostates. These mice developed intraepithelial hyperplasia by 22 weeks (**Fig. 5E**) and mPINs by 44 weeks (**Fig. 5F, J–L**). The prostate epithelium of TG mice was crowded with round to polygonal stratified nuclei, forming micropapillary projections and tufts (**Fig. 5E**). The acini were lined by a rim of basal cells (**Fig. 5F**). The areas of mPINs were easily identifiable and were characterized by prostatic acini containing intraluminal papillary structures lined by atypical cells with elongated nuclei exhibiting prominent nucleoli. Focally, the papillae merged into each other within the acini generating a cribiform pattern of growth (**Fig. 5J–L**). Dorsal lobe exhibited an increased number of small acini lined by cells containing nuclei exhibiting prominent nucleoli and the neoplastic acini were devoid of myoepithelial cells (**Fig. 5L**). We previously demonstrated that Ack1 regulates phosphorylation of androgen receptor [**26**] and tumor suppressor Wwox [25] in human prostate tumors. Neoplasia observed in PB-Ack1 mice could be due to the combined effect of Ack1 mediated AKT, AR and Wwox Tyr-phosphorylations. AR and Wwox Tyr-phosphorylations appear to be involved in late stage progression of prostate cancer to androgen-independence [26]. Ack1 mediated AKT Tyr176-phosphorylation and activation may be more proximal stage initiating processes in neoplastic progression that mimic or serve as an alternative to those of PTEN loss which has been prominently emphasized in other mouse models of prostate cancer [33].

### pTyr284-Ack1 and pTyr176-AKT Expressions Correlate with Breast Cancer Progression

**Figure 6:**
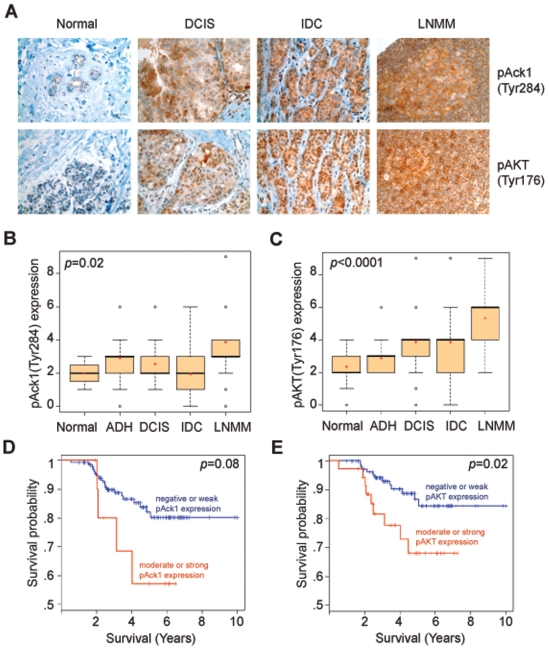
pTyr284-Ack1 and pTyr176-AKT expression in breast cancer. (**A**) TMA sections representing different breast cancer stages stained with pTyr284-Ack1 and pTyr176-AKT antibodies. (**B**) Box plots to summarize distributions of staining intensities for pTyr284-Ack1 in different stages of breast cancer. A significant increasing trend of intensity across progression stages was detected (Mantel-Haenszel 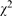 test, *p* = 0.02). The box has lines at the lower quartile (25%), median (50%), and upper quartile values (75%) while the red-cross within the circle marks the mean value. Whiskers extend from each end of the box to the most extreme values within 1.5 times the interquartile range from the ends of the box. The data with values beyond the ends of the whiskers, displayed with black circles, are potential outliers. (**C**) Box plots to summarize distributions of staining intensities for pTyr176-AKT in different stages of breast cancer. A significant increasing trend of intensity across progression stages was detected (Mantel-Haenszel 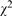 test, *p*<0.0001). (**D**) Kaplan–Meier analysis shows that individuals with breast cancer that have moderate to strong staining (>4) of pTyr284-Ack1 have a lower probability of survival (log rank test, *p = *0.08). (**E**) Kaplan–Meier analysis of the breast cancer patients that have moderate to strong staining (>4) of pTyr176-AKT have significantly lower probability of survival (log rank test, *p* = 0.02).

To examine the role of pTyr284-Ack1 and pTyr176-AKT in breast tumor progression, we performed an extensive tissue microarray analysis (TMA) of clinically annotated breast (n = 476) tumor samples. Tyr284 is the primary autophosphorylation site in Ack1, hence, phospho-Ack1(Tyr284) antibodies were used to assess Ack1 activation [27], [29]. Immunohistochemical analysis revealed that pTyr284-Ack1 and pTyr176-AKT were expressed in both membrane and nucleus (**Fig. S10A,B**). A significant increase in expression of pTyr284-Ack1 and pTyr176-AKT was seen when breast cancers from progressive stages were examined, i.e. normal to hyperplasia (ADH), ductal carcinoma *in situ* (DCIS), invasive ductal carcinoma (IDC) and lymph node metastatic (LNMM) stages (**Fig. 6A–C** and **Table 1**). In contrast to pTyr284-Ack1, the total Ack1 levels remained unchanged between normal and tumor samples (compare **Fig. S10D** and **E** with **F** and **G**). ANOVA results indicated that both pTyr284-Ack1 and pTyr176-AKT expression differed significantly among progression stages (*p*<0.0001). When using Tukey-Kramer method to examine all pairwise differences between different stages, the expression levels of pTyr284-Ack1 and pTyr176-AKT in LNMM were significantly higher than those of all the earlier tumor stages; the expression levels were significantly lower in the normal samples when compared to those of all the later stages except for hyperplasia (**Tables 2** and **3**). Kaplan-Meir analyses revealed that patients with high expression of pTyr284-Ack1 and pTyr176-AKT are at a higher risk for cancer-related deaths (**Fig. 6D, E** and **Table 4**). Furthermore, expression of pTyr284-Ack1 was significantly correlated with pTyr176-AKT *in situ* (Spearman rank correlation coefficient *ρ = *0.43, *p*<0.0001; **Fig. S10C**).

**Table 1:** The intensities of Tyr284-phosphorylated-Ack1 and Tyr176-phosphorylated-AKT for the trend analysis of breast cancer.

Protein	Statistics	Normal	ADH	DCIS	IDC	LNMM
**pTyr284-Ack1**	N	52	31	38	126	39
	Mean	2	2.9	2.55	1.94	3.87
	Median	2	3	2	2	3
	Std	0.714	1.3	1.25	1.41	2
	SE	0.1	0.23	0.20	0.13	0.32
	CI Low	1.8	2.43	2.14	1.7	3.22
	CI Upper	2.2	3.38	2.96	2.19	4.52
**pTyr176-AKT**	N	45	39	38	118	37
	Mean	2.36	2.9	3.97	3.86	5.32
	Median	2	3	4	4	6
	Std	0.8	0.79	1.96	2.17	1.93
	SE	0.12	0.13	0.32	0.2	0.32
	CI Low	2.11	2.64	3.22	3.46	4.68
	CI Upper	2.6	3.15	4.51	4.25	5.97

**Table 2:** P-values of Tukey-Kramer multiple comparisons (simultaneous inference) of pTyr284-Ack1 intensity levels between all pairs of stages for breast cancer.

pTyr284-Ack1	Normal	ADH	DCIS	IDC	LMM
**Normal**		0.0340*	0.3324	0.9992	<0.0001*
**ADH**			0.8313	0.0055*	0.3324
**DCIS**				0.1234	0.0004*
**IDC**					<0.0001*
**LMM**					

*indicate significance at 0.05 level.

**Table 3:** P-values of Tukey-Kramer multiple comparisons (simultaneous inference) of pTyr176-AKT intensity levels between all pairs of stages for breast cancer.

pTyr176-AKT	Normal	ADH	DCIS	IDC	LMM
**Normal**		0.6434	0.0016*	<0.0001*	<0.0001*
**ADH**			0.1276	0.0342*	<0.0001*
**DCIS**				1.0000	0.0049*
**IDC**					0.0002*
**LMM**					

*indicate significance at 0.05 level.

**Table 4:** Kaplan–Meier survival estimates by Tyr284-phosphorylated Ack1 and Tyr176-phosphorylated AKT intensities for breast cancer TMA samples.

Protein	Expression	No. of subjects	Event	Censored
pTyr284-Ack1	< = 4	133	14% (19)	86% (114)
pTyr284-Ack1	>4	11	36% (4)	64% (7)
pTyr176-AKT	< = 4	104	11% (11)	89% (93)
pTyr176-AKT	>4	36	25% (9)	75% (27)

## Discussion

**Figure 7:**
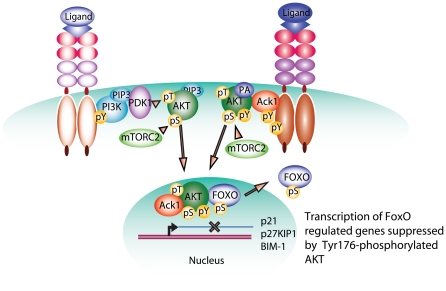
Tyr176-phosphorylation leads to AKT activation, a model. Our data demonstrates an alternate pathway of AKT activation wherein RTKs facilitate Ack1 phosphorylation at Tyr284 leading to its kinase activation. Ack1 could also be activated in some tumors by autoactivating somatic mutations, e.g. E346K. Activated Ack1 phosphorylates AKT at Tyr176 resulting in its binding to the anionic plasma membrane phospholipid PA. pTyr176-AKT localizes to the plasma membrane, where it is targeted by PDK1 and PDK2 (mTORC2 complex) for Thr308/Ser473 phosphorylations, respectively, leading to optimal AKT kinase activation. Activated AKT translocates to the nucleus, phosphorylates FoxO transcription factors to downregulate expression of FoxO target genes.

Our study indicates that cells employ multiple and possibly mutually exclusive mechanisms to activate AKT (**Fig. 7**). The reasons why RTKs would employ two distinct modes of AKT activation are not entirely clear. However, a fraction of AKT appears to utilize this alternative mode of activation in normal and prominently in cancerous cells. Our studies showed that even in the presence of PI3K inhibitor, ligand bound HER2/ErbB-2 or EGFR activated Ack1 which in turn Tyr-phosphorylated and activated AKT. AKT is frequently activated in pancreatic cancer which has been shown to be highly correlated to HER-2/*neu* overexpression [34]. Moreover, many of the pancreatic cell lines and tumors expressing activated AKT had retained wild-type PTEN [35], [36]. We noticed that PanIN, pancreatic adenocarcinoma and breast tumors of MMTV-*neu* mice exhibit significantly higher levels of pTyr284-Ack1 and pTyr176-AKT (unpublished data). Taken collectively, our data may explain AKT activation in those tumors that display amplification/activation of RTKs but have normal PI3K/PTEN levels. We propose that other tumors that possess somatic autoactivating mutations or amplification in non-receptor tyrosine kinases could use similar mechanisms for AKT activation [37].

Are there conditions when Tyr176 modification is not needed for AKT activation? Some of the conditions when Tyr176 phosphorylation of AKT is not required for AKT activation could be; 1) Presence of constitutively active *PIK3CA* mutations, observed in colorectal, glioblastomas, gastric breast and lung cancers [38]. 2) Loss of tumor suppressor PTEN resulting in increased levels of cellular PIP3, occur commonly in prostate cancer, endometrial cancer, and glioblastoma, among others [3]. 3) A rare somatic activating mutation, E17K in the pH domain which facilitates AKT recruitment to the membrane in PIP3-independent manner [13].

We have used the term AKT ‘translocation’ to indicate emergence of (cytosolic) AKT in the plasma membrane in response to growth factors. Our data (**Fig. 2B and 2C**) demonstrate that AKT in the plasma membrane is phosphorylated at Tyr 176 and mutation of this site in AKT abrogates appearance of AKT in the plasma membrane (**Fig. 2D**). Based on the evidence, our model (**Fig. 7**) suggests that as Ack1 signaling pathway is initiated at the plasma membrane by RTKs. Ack1 associates with growth factor-bound RTKs (via Mig6 homology domain in Ack1 carboxy terminal proline rich region) and is activated [25], [26], [39]. Ack1 is constitutively bound to AKT (**Fig. 1B** and **G**); Activated Ack1 directly phosphorylates AKT at Tyr176, thus facilitating accumulation of Tyr176-phosphorylated AKT at the plasma membrane. Tyr176-phosphorylated AKT preferentially binds PA, a plasma membrane phospholipid as opposed to unphosphorylated AKT (refer to **Fig. S8** for details). PH domain in AKT is a lipid binding domain and thus might be involved in the membrane binding of Tyr176-phosphorylated AKT. Collectively, our data suggests that Ack1 mediated AKT Tyr176-phosphorylation is driving this translocation process. Thus, although AKT Tyr176-phosphorylation and its migration to the plasma membrane is PIP3 independent, the recruitment of Tyr176 AKT in the plasma membrane may require a functional PH domain.

In contrast to AKT, pTyr176-AKT specifically binds the plasma membrane anionic phospholipid, PA (**Fig. S8**). Tyr176-phosphorylation could induce conformational changes in the AKT PH domain to enable binding to PA. The PH domain of Son of sevenless (SOS) and PX domains of p47^phox^ have previously been shown to possess a phosphoinositide-binding pocket and a second anion binding pocket which enables them to interact with PA facilitating plasma membrane recruitment [40], [41]. We speculate that AKT too might possess a masked anion binding pocket, and Tyr-phosphorylation induced conformational changes could unmask this pocket allowing it to bind PA.

In endogenous systems Ack1 associates with AKT2 albeit weakly as compared to AKT1 (**Fig. 1B**). AKT isoforms are differentially distributed among different cellular compartments [42] with majority of AKT1 in the cytosol, and AKT2 in the mitochondria. Additionally AKT2 protein appears to be not as abundant as AKT1 in MCF-7 and MEFs (**Fig. S1A**). Thus, weak interaction with AKT2 could be a combined outcome of differential cellular distribution and lower protein levels. However, our unpublised data demonstrates significant tyrosine phosphorylation of AKT2 upon coexpression of Ack1 and AKT2 in HEK293T cells, suggesting that both AKT1 and 2 are Ack1 substrates.

This study demonstrates that Tyr176-phosphorylation is sufficient for AKT membrane localization followed by PDK1/PDK2 mediated activation, defining the upstream Ack1 kinase activity as ‘PDK3’. We do not rule out the possibility that other tyrosine kinases may be able to target AKT for Tyr176-phosphorylation. Ack1 knockout mice are not currently available. However, when they are developed, they would allow us to investigate whether AKT can be phosphorylated at Tyr176 by other receptor or non-receptor tyrosine kinases in response to growth factors. Multiple non-receptor tyrosine kinases were earlier shown to increase AKT activity [43], [44], however, precise mechanism of AKT activation by any of the Tyr-modifications is not clear, nor is their role in initiation or progression of cancer. To our knowledge, this report provides the first demonstration for a role of Tyr-phosphorylated AKT in its compartmentalization, which allowed us to delineate its critical role in AKT kinase activation, its potential to initiate neoplasia in mouse prostates and promote disease progression in human breast cancers. Large numbers of tumors are reliant upon AKT activation for survival and growth making it an attractive target for molecular therapeutics [45]. The assay that was used during development of AKT inhibitors was primarily based on AKT Ser473-phosphorylation. Our data indicates that a new class of AKT inhibitors can be identified based on AKT Tyr176-phosphorylation. These novel inhibitors that block AKT membrane localization and activation could have major implications in cancer, diabetes and obesity research.

## Materials and Methods

### Ethics Statement

Mice breeding and colony maintenance was performed according to IACUC protocols approved in writing by University of South Florida (USF) and University of North Carolina at Chapel Hill Division of Research Integrity and Compliance. We used the breast TMA for our study for which we are exempt from IRB approval (once again written exemption) for this study, as no personal information about patients is sought.

### Materials

Mouse embryo fibroblasts derived from AKT1, AKT2 and AKT1&2 knockout mice were obtained from Dr. Morris J. Birnbaum, University of Pennsylvania, Philadelphia. Human Embryonic Kidney cell line 293T, normal prostate cell line RWPE and MCF-7 cells were obtained from the American Type Tissue Culture Collection. Ack1 mAb (A11), alpha-tubulin (TU-O2), Actin (I-19), EGFR(1005), pTyr(PY20)HRP conjugate antibodies purchased from Santacruz; Anti-phospho-Ack1 (Tyr284, Upstate); phospho-AKT (Thr308, C31E5E), phospho-AKT (Ser473, D9E), AKT [20] (C67E7 Rabbit mAb), AKT1(C73H10 Rabbit mAb), AKT2(5B5 Rabbit mAb), phospho-AKT (Ser473, 193H12) Rabbit mAb Alexa Fluor 647 conjugate, HA-Tag (6E2) Mouse mAb Alexa Fluor 488, phosphoHistone H3-Serine10 Alexa Fluor 647 conjugate antibodies and LY294002 purchased from Cell Signaling, NaKATPase (ab7671, Abcam), c-erbB-2/Her2/neu Ab-2 (Clone 9G6.10) (Thermo Scientific) antibodies, Ku70 (N3H10, Neomarkers), were purchased from the respective companies. Site directed mutagenesis was performed to generate the AKT(Y176F), AKT(R25C), myrAKT (Y176F), Ack1(E346K), Ack1(R34L), Ack1(R99Q) and Ack1 (H409I) constructs according to the manufacturer's protocol (Promega Inc.). EGFP-E346K and DsRed2-N1-AKT (WT and Y176F) were generated by subcloning E346K and AKT cDNAs into the pEGFP-N1 and pDsRed2-N1 (Clontech) vectors respectively. Control and Ack1 siRNAs were generated by custom synthesis (Qiagen) and the sequences have been described previously [26]. PI3K siRNAs (SC39127) and antibodies were purchased from Santacruz.

### AKT Phospho-Site Determination Using Mass Spectrometry

293T cells co-expressing activated Ack and HA-tagged AKT were lysed in receptor lysis buffer (RLB) containing 25 mmol/L Tris (pH 7.5), 225 mmol/L NaCl, 1% Triton X-100, 1 mmol/L DTT, 10% glycerol, phosphatase inhibitors (10 mmol/L NaF, 1 mmol/L Na_2_VO_4_), and protease inhibitor mix (Roche). Following immunoprecipitation with HA-beads (E6779, Sigma, St. Louis, MO), purified AKT was subjected to SDS PAGE electrophoresis and the gel was stained Coomassie Brilliant Blue-R250(BioRad). A prominent band of ∼59 kDa was excised, washed once with water and twice with 50 mM ammonium bicarbonate in 50% aqueous methanol. Proteins were reduced and alkylated with 2 mM Tris(2-carboxyethyl)phosphine hydrochloride (TCEP) (Sigma, St. Louis, MO) and 20 mM iodoacetamide (GE Healthcare, Pittsburgh, PA), respectively. Samples were digested overnight with modified sequencing grade trypsin (Promega, Madison, WI), Glu-C (Worthington, Lakewood, NJ), or chymotrypsin (Roche, Switzerland). Peptides were extracted from the gel slices, phosphopeptides were enriched using IMAC spin columns (Pierce, Rockford, IL) or TiO_2_ Mono tip (GL Science, Japan). A nanoflow liquid chromatograph (Ultimate3000, LC Packings/Dionex, Sunnyvale, CA) coupled to an electrospray hybrid ion trap mass spectrometer (LTQ Orbitrap, Thermo, San Jose, CA) was used for tandem mass spectrometry peptide sequencing experiments. Peptides were separated with a C18 reverse phase column (LC Packings C18Pepmap) using a 40 min gradient from 5%B to 50%B (B: 90% acetonitrile/0.1% formic acid). The flow rate on the analytical column was 300 nl/min. Five tandem mass spectra were acquired for each MS scan using 60 sec exclusion for previously sampled peptide peaks (Spray voltage 2.3 kV, 30% normalized collision energy, scanning m/z 450–1,600). Sequences were assigned using Sequest (Thermo) and Mascot (www.matrixscience.com) database searches against SwissProt protein entries of the appropriate species. Oxidized methionine, deamidation, carbamidomethyl cysteine, and phosphorylated serine, threonine and tyrosine were selected as variable modifications, and as many as 3 missed cleavages were allowed. The precursor mass tolerance was 1.08 Da and MS/MS mass tolerance was 0.8Da. Assignments were manually verified by inspection of the tandem mass spectra and coalesced into Scaffold reports (www.proteomesoftware.com).

### Generation and Purification of pTyr176-AKT Phospho-Antibody

Two AKT peptides coupled to immunogenic carrier proteins were synthesized.

The phosphopeptide: Ac-ATGRY[pY]AMKIL-Ahx-C-amide

The non-phospho peptide: Ac-ATGRYYAMKIL-Ahx-C-amide

Two rabbits were immunized twice with phosphopeptide, several weeks apart, and enzyme-linked immunosorbent assay was performed to determine the relative titer of sera against phosphorylated and nonphosphorylated peptides. The titer against phosphorylated peptides (1∶40,000) was much greater than nonphosphorylated peptide (1∶2700). The sera were affinity-purified. In brief, two antigen-affinity columns were used to purify the phospho-specific antibodies. The first column was the non-phosphopeptide affinity column. Antibodies recognizing the non-phospho residues of the peptide bound to the column and were eluted as pan-specific antibodies. The flow-through fraction was collected and then applied to the second column, the phospho-peptide column. Antibodies recognizing the phospho-residue bound to the column which was eluted as phospho-specific antibodies. The purified antibodies were extensively characterized for various applications e.g. Western blotting and immunohistochemistry.

### Cell Fractionation, Immunoprecipitations and Kinase Assay

Membrane and cytosolic fractionation was performed using kit from Biovision. The nuclear/cytoplasmic fractionation was performed using protocol from Abcam. For immunoprecipitations, cells were lysed in receptor lysis buffer (RLB) containing 25 mmol/L Tris (pH 7.5), 500 mmol/L NaCl, 1% Triton X-100, 10% glycerol, phosphatase inhibitors (10 mmol/L NaF, 1 mmol/L Na_2_VO_4_), and protease inhibitor mix (Roche). For co-immunoprecipitation, cells were lysed in buffer containing 25 mmol/L Tris (pH 7.5), 225 mmol/L NaCl, 1% Triton X-100, 10% glycerol, phosphatase inhibitors (10 mmol/L NaF, 1 mmol/L Na_2_VO_4_), and protease inhibitor mix (Roche). The kinase assay was performed using kit from Calbiochem.

### Purification, *In Vitro* Binding and Phosphorylation Assay

GST-Ack1 was purified using method described earlier [31]. HEK293T cells were transfected with HA-tagged Ack1, AKT, Y176F mutant of AKT and FLAG-tagged AR; 48 hours post-transfection cell were lysed in RLB buffer. Lysates were incubated with HA beads (Sigma) for 2 h, followed by washing with RLB buffer and elution in PBS containing HA or FLAG peptide (2 mM) on ice. Purity of preparation was confirmed by coomassie blue staining of gel. For the *in vitro* binding assay, 50 nM of purified Ack and AKT were incubated in modified RLB (mRLB) containing 25 mM Tris (pH 7.5), 175 mM NaCl, 1% Triton X-100, 10% glycerol, and protease inhibitor mix at room temperature. After 30 mins, anti-Ack1 antibodies and Protein-A-sepharose beads were added, incubated with shaking at 4°C for overnight. Beads were washed thrice with mRLB buffer. Bound protein complex was dissociated from beads by boiling in SDS sample buffer and assessed by gel electrophoresis and detection by immunoblotting with anti-AKT antibody. In a control experiment, immunoprecipitation was done using non-specific IgG. For *in vitro* phosphorylation of AKT by Ack1, 50 nM of purified Ack1 and AKT were incubated in kinase buffer contained 20 mmol/L HEPES (pH 7.5), 150 mM NaCl, 10 mmol/L MgCl_2_, 0.1 mmol/L Na_2_VO_4_, 0.5 mmol/L DTT, 0.25 mmol/L ATP for 1 hour at 30°C. The reaction was stopped by adding sample buffer and reaction was assessed by gel electrophoresis and detection by immunoblotting with antibodies as shown.

### Quantitative RT-PCR

All RT reactions were done at the same time so that the same reactions could be used for all gene studies. For the construction of standard curves, serial dilutions of pooled sample RNA were used (50, 10, 2, 0.4, 0.08, and 0.016 ng) per reverse transcriptase reaction. One “no RNA” control and one “no Reverse Transcriptase” control were included for the standard curve. Three reactions were performed for each sample: 10 ng, 0.8 ng, and a NoRT (10 ng) control. Real-time quantitative PCR analyses were performed using the ABI PRISM 7900HT Sequence Detection System (Applied Biosystems). All standards, the no template control (H_2_O), the No RNA control, the no Reverse Transcriptase control, and the no amplification control (Bluescript plasmid) were tested in six wells per gene (2 wells/plate x 3 plates/gene). All samples were tested in triplicate wells each for the 10 ng and 0.8 ng concentrations. The no RT controls were tested in duplicate wells. PCR was carried out with SYBR Green PCR Master Mix (Applied Biosystems) using 2 µl of cDNA and the primers (**Table 5**) in a 20-µl final reaction mixture: *Actin*: 300/300 nM; *p21*: 300/300 nM; *p27Kip1*-1∶300/300 nM; *p27Kip1*-2: 300/300 nM; *FASL*-2: 300/300 nM; *GADD45*-1: 300/300 nM; *GADD45*-2: 300/300 nM; *BIM*: 100/100 nM; *HPRT1*: 100/100 nM. After 2-min incubation at 50°C, AmpliTaq Gold was activated by a 10-min incubation at 95°C, followed by 40 PCR cycles consisting of 15 s of denaturation at 95°C and hybridization of primers for 1 min at 55°C. Dissociation curves were generated for each plate to verify the integrity of the primers. Data were analyzed using SDS software version 2.2.2 and exported into an Excel spreadsheet. The actin data were used for normalizing the gene values; i.e., ng gene/ng actin per well.

**Table 5:** Primer sequences for qRT-PCR.

Primer	Sequence
*p27Kip1* Fwd	TCAAACGTGAGAGTGTCTAACG
*p27Kip1* Rev	CCGGGCCGAAGAGATTTCTG
*p21* Fwd	TGTTCCGCACAGGAGCAA
*p21* Rev	TGAGCGCATCGCAATCA
*Bim* Fwd	CCCGGAGATACGGATTGCAC
*Bim* Rev	GCCTCGCGGTAATCATTTGC
*Gadd45* Fwd	AGACCGAAAGGATGGACACG
*Gadd45* Rev	TGACTCCGAGCCTTGCTGA
*HPRT* Fwd	CACAGGACTAGAACACCTGC
*HPRT* Rev	GCTGGTGAAAAGGACCTCT
*ACTB* Fwd	GTGGGCATGGGTCAGAAG
*ACTB* Rev	TCCATCACGATGCCAGTG

### Fluorescence Microscopy

For cellular localization studies, NIH3T3 cells grown on coverslips were transfected at 50% confluency. Cells were fixed with 4% paraformaldehyde in PBS for 10 min, washed with PBS. Coverslips with fixed cells were mounted on slides in Vectashield mounting medium with DAPI (Vector Laboratories), and red (dsRed2-N1AKT) or green (EGFP-346K) fluorescence was detected using a Zeiss Automated Upright Fluorescent Microscope and charge-coupled device (CCD) camera with appropriate filters. Zeiss Axiovision software was used for image viewing and processing.

### Ack1 Transgenic (TG) Mice

For *in vivo* expression of Ack1, Myc-epitope-tagged construct was generated in two steps. First, PCR was performed using ARR2PB promoter region (provided by UNC Mouse Core Facilities) as the template, which was subcloned in pTG1 vector. In the second step, a PCR product was generated using activated Ack1(L487F) mutant (Mahajan, 2005 #12) as the template and the reverse primer encoding a Myc-tag. The caAck PCR product (1 to 787 aa) was digested and was inserted into the pTG1 vector downstream of a sequence coding Globin intron and upstream of a SV40 polyA site (the schematic is shown in **Fig. 5A**). The construct was sequenced. The ARR2PB-Ack1 plasmid was digested with HindIII and BamHI and a 4Kb linear DNA fragment was gel purified and microinjected into fertilized C57B6 mouse eggs, which were then surgically transplanted into a pseudo-pregnant female. Transgenic founders were screened by PCR using genomic DNA isolated from tail snips. The prostate specific expression was assessed by immunoprecipitation with Myc-antibodies followed by immunoblotting with pTyr-antibodies (**Fig. 5B**). TG and WT mice were sacrificed at various time points for removal of prostate followed by lysate preparation and immunoblotting (**Fig. 5C**). Prostates from transgenic mice were dissected using a dissection microscope, fixed in 10% buffered formalin and embedded in paraffin. Sections were stained with haematoxylin and eosin and stained slides were evaluated by pathologist (R.W.E and A.S.L.).

### Flow Cytometry Analysis

AKT 1&2KO MEFs transfected with either the AKT WT or 176 mutant constructs were serum starved 24 h post-transfection. Cells were either untreated or treated with EGF for 15 minutes and harvested. Cells were singly or doubly stained with antibodies; AKT Ser473 conjugated to Alexa 647 and HA tag conjugated to Alexa 488 according to the manufacturer's protocol (Cell Signaling). Briefly, cells were resuspended in 1X Phosphate Buffered Saline (PBS) to which paraformaldehyde was added to a final concentration of 4%. Cells were fixed at 37°C for 10 min and chilled on ice for 1 min. The fixative was removed after centrifugation at 1500 rpm for 5 min. Cells were resuspended in ice cold 100% methanol and incubated on ice for 30 min and stored at -20C in 90% methanol. One million cells from each sample were rinsed with 2 ml of 1XPBS containing 0.5% BSA by centrifugation and resuspended in 90 µl of incubation buffer per assay tube for 10 min. 10 µl of conjugated antibody was added to the assay tube and incubated for 60 min in the dark at room temperature. The cells were rinsed twice with the incubation buffer by centrifugation and resuspended in 0.5 ml PBS and accquired on FACS calibur and analyzed by the FlowJo software.

### Tissue Microarray (TMA) Analysis

For assessment of pTyr284-Ack1 and pTyr176-AKT expression in breast cancer, immunohistochemistry was carried out on two high-density TMAs (n = 476 cores) containing samples of normal breast tissue, atypical ductal hyperplasia (ADH), ductal carcinoma *in situ* (DCIS), invasive ductal carcinoma (IDC), lymph node macro metastasis (LNMM). Four µm sections were cut with Leica microtome (Leica Microsystems Inc, Bannockburn, IL) and transferred to adhesive-coated slides. The tissue array slides (4 slides including 2 test duplicate slides, and positive and negative controls) were stained for pTyr284-Ack1 and pTyr176-AKT using respective rabbit polyclonal antibodies. The slides were dewaxed by heating at 55 Celsius for 30 min and by three washes, 5 min each, with xylene. Tissues were rehydrated by series of 5 min washes in 100%, 95%, and 80% ethanol and distilled water. Antigen retrieval was performed by heating the samples at 95° Celsius for 30 min in 10 mmol/L sodium citrate (pH 6.0). After blocking with universal blocking serum (DAKO Diagnostic, Mississauga, Ontario, Canada) for 30 min, the samples were then incubated with rabbit polyclonal pTyr284-Ack1 antibody (1∶300 dilution; Milipore) and rabbit polyclonal phospho-AKT antibody (1∶25 dilution) at 4° Celsius overnight. The sections were incubated with biotin-labeled secondary and streptavidin-peroxidase for 30 min each (DAKO Diagnostic). The samples were developed with 3,3′-diaminobenzidine substrate (Vector Laboratories, Burlington, Ontario, Canada) and counterstained with hematoxylin. Following standard procedures the slides were dehydrated and sealed with cover slips. Negative controls were included by omitting pTyr284-Ack1/pTyr176-AKT antibody during primary antibody incubation. The phospho-AKT/Ack1 antibodies were extensively validated for immunohistochemistry studies. MCF7 cells treated with heregulin and RWPE cells treated with EGF ligand (or no ligand) were fixed, paraffin imbedded, sectioned and used for antibody validation. Further, MEF1&2KO cells transfected with activated Ack1 and AKT were also used to validate antibodies. The pTyr284-Ack1 and pTyr176-AKT staining in paraffin embedded tissues were examined in a blinded fashion by two independent pathologists (A.L. and D.C.). If needed, a consensus score was reached for each specimen. The positive reactions were scored into four grades according to the intensity of staining: 0, 1+, 2+ and 3+. The percentages of pTyr176-AKT positive cells were also scored into four categories: 0 (0%), 1+ (1–33), 2+ (34–66), 3+ (more than 66%). The product of the intensity and percentage scores was used as a final staining score.

### Statistical Analysis

The Mantel-Haenszel 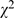 test was performed to examine if there is an increasing trend for pTyr284-Ack1 and pTyr176-AKT with respect to different progression stages of breast or pancreatic cancer. The ordinal intensity levels of pTyr284-Ack1 and pTyr176-AKT 0, 1, 2, 3, 4, 6, 9 were pooled into 6 levels (as 0, 1, 2, 3, 4, and 6 and above) to accommodate the rare observations in the highest intensity level in most stages. Analysis of variance was performed to examine whether the expression levels of pTyr284-Ack1 and pTyr176-AKT differ among different tumor stages. Boxplots were used to summarize the intensity distribution at each progression stage. Furthermore, Tukey-Kramer method was performed to examine between which pairs of stages the expression levels are different. This post-hoc procedure adjusts for all pairwise comparisons and simultaneous inference. When more than one sample was obtained from a patient, the intensity of the most progressed stage was used for the analysis. Correlation between pTyr284-Ack1 and pTyr176-AKT was explored using Spearman ranked correlation analysis. The association of the expression levels of pTyr284-Ack1 and pTyr176-AKT and the overall survival of patients were assessed using the Kaplan–Meier method. For breast cancer data, there were 144 individuals with available pTyr284-Ack1 staining and survival information while there were 140 individuals with available pTyr176-AKT staining and survival information. For pancreatic cancer data, there were 83 individuals with available pTyr284-Ack1 staining and survival information while there were 76 individuals with available pTyr176-AKT staining and survival information. Statistical differences between the groups were determined using log-rank test.

## Supporting Information

Figure S1 AKT is Tyr-phosphorylated by Ack1 *in vitro*. (A) AKT MEF KO1, KO2 and KO1&2s lack respective AKT isoforms. Equal amounts of MEFs protein lysates were subjected to IB as indicated. MCF-7 cell lysate was used as control. (B) Purification of Ack1 and AKT. HA-tagged Ack1 and AKT were expressed in HEK293T cells, lysed and incubated with HA-beads. Followed by extensive washing, proteins were eluted using HA-peptide (2nM, 1 hour) and assessed by SDS-PAGE and Coomassie Brilliant Blue-R250(BioRad) staining. (C) *In vitro* binding assay. Equimolar amounts of purified Ack1 and AKT proteins were incubated for 30 min, complex was immunoprecipitated with Ack1 (lanes 2–5) or IgG (lane#6) antibodies followed by IB with anti-AKT antibodies (top panel). About 6.35% of total AKT was in complex with Ack1. (D) *In vitro* phosphorylation of purified AKT by Ack1. Equimolar amounts of purified Ack1 and AKT proteins were incubated in kinase buffer for 1 hour at 370C and reaction mix was subjected to IB with pTyr176-AKT (top panel), pTyr (2nd and 3rd panels), AKT (4th panel) and Ack1 (bottom panel) antibodies. (E) Schematic representation of GST-Ack1 construct. FLAG-tagged AR expressed in HEK293 cells and GST-tagged Ack1 was expressed in DH5 cells. Purified GST-Ack1 (right panel) and FLAG-AR (left panel) were assessed by SDS-PAGE followed by Coomassie staining. (F) *In vitro* binding assay. Equimolar amounts of purified HA-AKT or FLAG-AR proteins were incubated with GST-Ack1 bound to beads for overnight, beads were washed followed by IB with anti-FLAG/HA antibodies (top panel). Lower panels show IB with FLAG/HA (2nd panel) and GST (bottom panel) antibodies.
(0.05 MB PDF)

Figure S2 Tyr176-phosphorylated AKT sample also contains Thr308 and Ser473 phosphorylated AKT. (A) Activated Ack1 (caAck) and HA-tagged AKT were coexpressed in HEK293T cells followed by IP with HA-beads. IP AKT was subjected to SDS-PAGE electrophoresis and the gel was stained Coomassie. A prominent band of ∼59 kDa corresponding to AKT is seen which was excised and subjected to mass spectrometry as described in methods section. The upper ∼113 kDa band corresponds to caAck1 that bound to AKT. (B) Purified AKT peptide preparation that lead to the identification of pTyr176-AKT was assessed for other phosphorylation events. A peptide was detected at 21.12 mins in the total ion chromatogram with mass-to-charge ratio 918.43, which represents an error of 1.0 ppm (C). (D) The tandem mass spectrum matched the sequence, FGLCKEGIKDGATMKpTFC indicating that Thr308 in AKT was phosphorylated; the detection of the phosphothreonine y3 is consistent with this localization. (E) Another peptide was detected at 23.72 mins in the total ion chromatogram with mass-to-charge ratio 944.93, which represents an error of 0.99 ppm (F). (G) The tandem mass spectrum matched the sequence, ERRPHFPQFpSYSASGTA indicating that Ser473 in AKT was phosphorylated; the detection of b8, b9, y7 and y8 is consistent with this localization.
(0.08 MB PDF)

Figure S3 AKT Tyr176-phosphorylation affects the loop harboring Ser473. (A) Residues Tyr176 and Ser473 are located in regions with increased conformational flexibility. The backbone of AKT1 is color-traced according to crystallographic B-factors from blue (20 Angstrom, less flexible) to red (76 Angstrom, highly flexible). (B) B-factor plot of all C-alpha atoms. The average main chain B-factor is 36 Angstrom (dashed horizontal line). (C) AKT Tyr176-phosphorylation induces substantial conformational changes of residues in its vicinity. Electrostatic interactions could be established with Arg174 and/or Lys214 while electrostatic repulsion and/or steric hindrance (due to the bulky phosphate group) may affect Glu169 and Tyr215. This could lead to a shift of the beta-strand flanking the c-terminal portion of the loop harboring Ser473, in turn causing structural alterations of this residue.
(0.07 MB PDF)

Figure S4 Kinase domain of Ack1 interacts with AKT PH domain/Tyr176 in kinase domain. (A) Schematic representation of wild type AKT, Y176F point mutant and deletion constructs. Site-directed mutagenesis of AKT was performed to generate the tyrosine to phenylalanine, Y176F, point mutant. PH, Pleckstrin homology domain; Kinase, Kinase domain and CT, Carboxy Terminal regulatory region. Schematic representation of Ack1 and deletion constructs. SAM, Sterile alpha motif; Kinase, kinase domain; SH3, Src homology domain 3; C, Cdc42 Rac interactive binding domain. (B) Flow cytometry of AKT 1&2KOMEFs, expressing HA-AKT and/or HA-Y176F. Top left panel indicates mock transfected cells stained with AKT-Ser473 antibody conjugated to Alexa 647 (untreated: 0.1%). Bottom left panel shows percentage of cells with AKT Ser473-phosphorylation upon EGF stimulation (15.2%). Right top and bottom panels show percentage of cells expressing HA-AKT (23%) or HA-Y176F (31%), respectively, in cells stained with anti-HA antibody conjugated to Alexa 488. (C) MEF1&2KO cells were co-transfected with HA-tagged AKT deletions and caAck1. The lysates were IP using HA antibodies followed by IB with pTyr antibodies (top panel). Lower panel show IP using HA antibodies followed by IB with AKT antibodies. Bottom panel show IB of the lysate with Ack1 antibodies. (D) HEK293 cells were co-transfected with HA-tagged AKT deletions and myc-tagged caAck. The lysates were IP using Myc antibodies followed by IB with HA antibodies (top panel). Lower panels are as described above. (E) MEF1&2KO cells were transfected with myc-tagged Ack1 deletions and HA-tagged AKT. The lysates were IP using Myc antibodies followed by IB with AKT antibodies (top panel). Lower panels show IB with Myc and AKT antibodies.
(0.08 MB PDF)

Figure S5 Somatic autoactivation of Ack1. (A) Schematic representation of Ack1 and various point mutants identified in the COSMIC database. Site-directed mutagenesis of Ack1 was performed to generate four HA-tagged point mutants. SAM, Sterile alpha motif; Kinase, kinase domain; SH3, Src homology domain 3; C, Cdc42 Rac interactive binding domain; Proline, Proline rich domain; UBA, Ubiquitin binding domain. (B) E346K mutation results in Ack1 autoactivation leading to AKT activation. MEF1&2KO cells were transfected with Ack1 mutants and the lysates were IP using anti-HA antibodies followed by IB with pTyr antibodies (top panel). Lower panels show IB with indicated antibodies. (C) E346K mutant Ack1 interacts with and Tyr-phosphorylates AKT. 293T cells were co-transfected with HA-tagged Ack1 point mutants. Equal amounts of protein lysates were subjected to IP using HA antibodies. IB with AKT antibodies revealed formation of activated Ack1(E346K)/endogenous AKT complex (top panel). (D) HEK293T cells were transfected with HA-tagged E346K, caAck or kdAck (K158R) mutants. Lysates were subjected to IP using anti-HA (top panel) antibodies followed by IB with pTyr284-Ack1 antibodies. Lower panels show IB with indicated antibodies. (E) E346K or caAck mediated AKT Tyr-phosphorylation leads to AKT kinase activation. HEK293T cells were co-transfected with E346K or myc-tagged caAck and AKT or Y176F mutant. Lysates were subjected to IP using anti-myc (top panel) and anti-Ack1 (second panel) antibodies followed by IB with pTyr antibodies. The same lysates were processed for kinase assay shown in S6F. (F) Ack1 autoactivation leads to AKT kinase activation. As described in S6E, lysates were IP with HA-antibodies, followed by AKT kinase assay. Low levels of Ack1 kinase activity in vector transfected cells was treated as zero and increased kinase activity (in percentage) over the vector expressing cells is shown.
(0.05 MB PDF)

Figure S6 Generation and validation of pTyr176-AKT phospho-antibodies. (A) EGF and heregulin treatment results in AKT Tyr176-phosphorylation. RWPE, normal prostate epithelial cells were treated with EGF (10 ng/ml,10 mins) and heregulin (10 ng/ml, 35 mins) ligand, equal amounts of protein lysates were subjected to immunoblotting as indicated. pTyr176-antibodies specifically recognizes endogenous Tyr-phosphorylated AKT following treatment with ligands. (B) Ack1 activation lead to AKT Tyr176-phosphorylation. 293T cells were co-transfected with myc-tagged caAck or kdAck and AKT or Y176F mutant. Equal amounts of protein lysates were subjected to immunoblotting with pTyr176-AKT antibodies. The pTyr176-antibodies recognize only the pTyrAKT (lane 2), but not the Y176F point mutant (lane 4). (C-J) Tyr176-phosphorylated AKT localizes at plasma membrane. NIH3T3 cells were co-transfected with EGFP-E346K mutant of Ack1 and dsRed2-N1-AKT (D-F) or dsRed2-N1-Y176F-AKT (G-J) DNAs overnight. Cells were serum starved, fixed and visualized by fluorescence microscopy. AKT but not Y176F mutant was localized to the plasma membrane in activated Ack1(E346K) expressing cells.
(0.07 MB PDF)

Figure S7 Tyr176-phosphorylation of mutant AKT (R25C) that inefficiently binds phosphatidyl-inositol 3,4, 5-triphosphate. (A) MEF1&2KO cells were transfected with activated Ack and AKT followed by LY294002 (10 µM) for 1 h. Cell lysates were fractionated and subjected to immunoblotting with indicated antibodies. AKT Ser473 phosphorylation in membrane fraction was unaffected by LY294002 treatment suggesting Ack1 mediated AKT activation is not dependent upon PI3K activity. (B) Schematic representation of wild type AKT and R25C point mutant constructs. Site-directed mutagenesis of AKT was performed to generate the R25C point mutant. PH, Pleckstrin homology domain; Kinase, Kinase domain and CT, Carboxy Terminal regulatory region. (C) MEF1&2 KO cells were transfected with empty vector or caAck and HA-tagged AKT or R25C mutant DNAs. Serum starved (18 h) cells were treated with EGF (10 ng/ml, 15 mins). The lysates were subjected to immunoprecipitation with anti-HA (top panel) or anti-Ack1 (second panel) antibodies followed by immunoblotting with pTyr antibodies. (D) MEF1&2 KO cells were transfected with empty vector or caAck and HA-tagged AKT or R25C mutant DNAs. Serum starved (18 h) cells were treated with EGF (10 ng/ml, 15 min). Cell lysates were fractionated and subjected to immunoblotting.
(0.05 MB PDF)

Figure S8 Tyr-phosphorylated AKT binds to phosphatidic acid. Protein-phospholipid overlay assay was performed using nitrocellulose membranes spotted with 100 pmol of different phospholipids. (A-C, F,G) Cells transfected with vector or activated Ack1 and AKT or Y176F were lysed and immunoprecipitated with pTyr-beads followed by elution with phenylphosphate. The eluted Tyr-phosphorylated proteins were incubated with phospholipid blots overnight at 4°C. Blots were extensively washed and bound proteins were detected with (A, B and F) pTyr176-AKT and (C and G) AKT antibodies. (D and E) Cells expressing HA-tagged AKT (D) and Y176F mutant (E) were lysed and immunoprecipitated with HA-beads followed by elution with HA peptide. The eluate was incubated with phospholipid blot and bound protein was detected with AKT antibodies. The pTyr176-AKT bound to phosphatidic acid, in contrast, AKT protein primarily binds to phosphatidyl-inositol 3,4,5-triphosphate (PIP3). (H) HA-peptide and phenylphosphate eluates were immunoblotted with antibodies shown to confirm presence of desired proteins.
(0.03 MB PDF)

Figure S9 Tyr176 phosphorylated AKT is enriched in the nucleus. (A) MCF-7 cells were serum starved (24 h) and treated with heregulin (30 ng/ml) for indicated times. Cell lysates were fractionated into nuclear and cytoplasmic fractions. Equal amounts of protein from these two fractions were subjected to immunoblotting with indicated Abs. Activated Ack1 mediated Tyr176 phosphorylated AKT is enriched in the nucleus 45 mins after heregulin treatment. The mobility of pTyr176-AKT is affected due to difference in the salt concentrations of nuclear (300 mM NaCl) and cytoplasmic fractions (10 mM KCl) (top panel). (B) MEF1&2KO cells were transfected with HA-tagged myr-AKT or myr-Y176F, equal amounts of protein lysates were subjected to immunoblotting as indicated. The myristoylated-AKT exhibits high levels of AKT activation, as seen by Thr308-phosphorylation.
(0.03 MB PDF)

Figure S10 Staining of tumor samples with Tyr284-phosphorylated-Ack1 and Tyr176-phosphorylated-AKT antibodies. Representations of Tyr284-phosphorylated-Ack1 (A) and Tyr176-phosphorylated-AKT (B) staining of IDC, which show intense staining in nuclei and membrane. (C) Expression levels between Tyr284-phosphorylated-Ack1 and Tyr176-phosphorylated-AKT expression were significantly correlated in breast tumors (Spearman rank correlation coefficient rho = 0.43, p<0.0001). (D–G) Breast samples stained with Ack1 and pAck1(Tyr284) antibodies. Basal levels of Ack1 expression were seen in both normal and tumor samples (D, E), however, significant increase in pAck1(Tyr284) staining was seen in tumor samples as contrast to normal breast sample (compare F and G).
(0.10 MB PDF)
