# Supplementary material for: Ack1 Mediated AKT/PKB Tyrosine 176 Phosphorylation Regulates Its Activation
Source: PLoS One. 2010 Mar 19;5(3):e9646. doi: 10.1371/journal.pone.0009646 (PMC2841635; doi:10.1371/journal.pone.0009646)
Supplement: Figure S1 — AKT is Tyr-phosphorylated by Ack1 in vitro. (A) AKT MEF KO1, KO2 and KO1&2s lack respective AKT isoforms. Equal amounts of MEFs protein lysates were subjected to IB as indicated. MCF-7 cell lysate was used as control. (B) Purification of Ack1 and AKT. HA-tagged Ack1 and AKT were expressed in HEK293T cells, lysed and incubated with HA-beads. Followed by extensive washing, proteins were eluted using HA-peptide (2nM, 1 hour) and assessed by SDS-PAGE and Coomassie Brilliant Blue-R250(BioRad) staining. (C) In vitro binding assay. Equimolar amounts of purified Ack1 and AKT proteins were incubated for 30 min, complex was immunoprecipitated with Ack1 (lanes 2–5) or IgG (lane#6) antibodies followed by IB with anti-AKT antibodies (top panel). About 6.35% of total AKT was in complex with Ack1. (D) In vitro phosphorylation of purified AKT by Ack1. Equimolar amounts of purified Ack1 and AKT proteins were incubated in kinase buffer for 1 hour at 370C and reaction mix was subjected to IB with pTyr176-AKT (top panel), pTyr (2nd and 3rd panels), AKT (4th panel) and Ack1 (bottom panel) antibodies. (E) Schematic representation of GST-Ack1 construct. FLAG-tagged AR expressed in HEK293 cells and GST-tagged Ack1 was expressed in DH5 cells. Purified GST-Ack1 (right panel) and FLAG-AR (left panel) were assessed by SDS-PAGE followed by Coomassie staining. (F) In vitro binding assay. Equimolar amounts of purified HA-AKT or FLAG-AR proteins were incubated with GST-Ack1 bound to beads for overnight, beads were washed followed by IB with anti-FLAG/HA antibodies (top panel). Lower panels show IB with FLAG/HA (2nd panel) and GST (bottom panel) antibodies. (0.05 MB PDF) [file pone.0009646.s001.pdf]

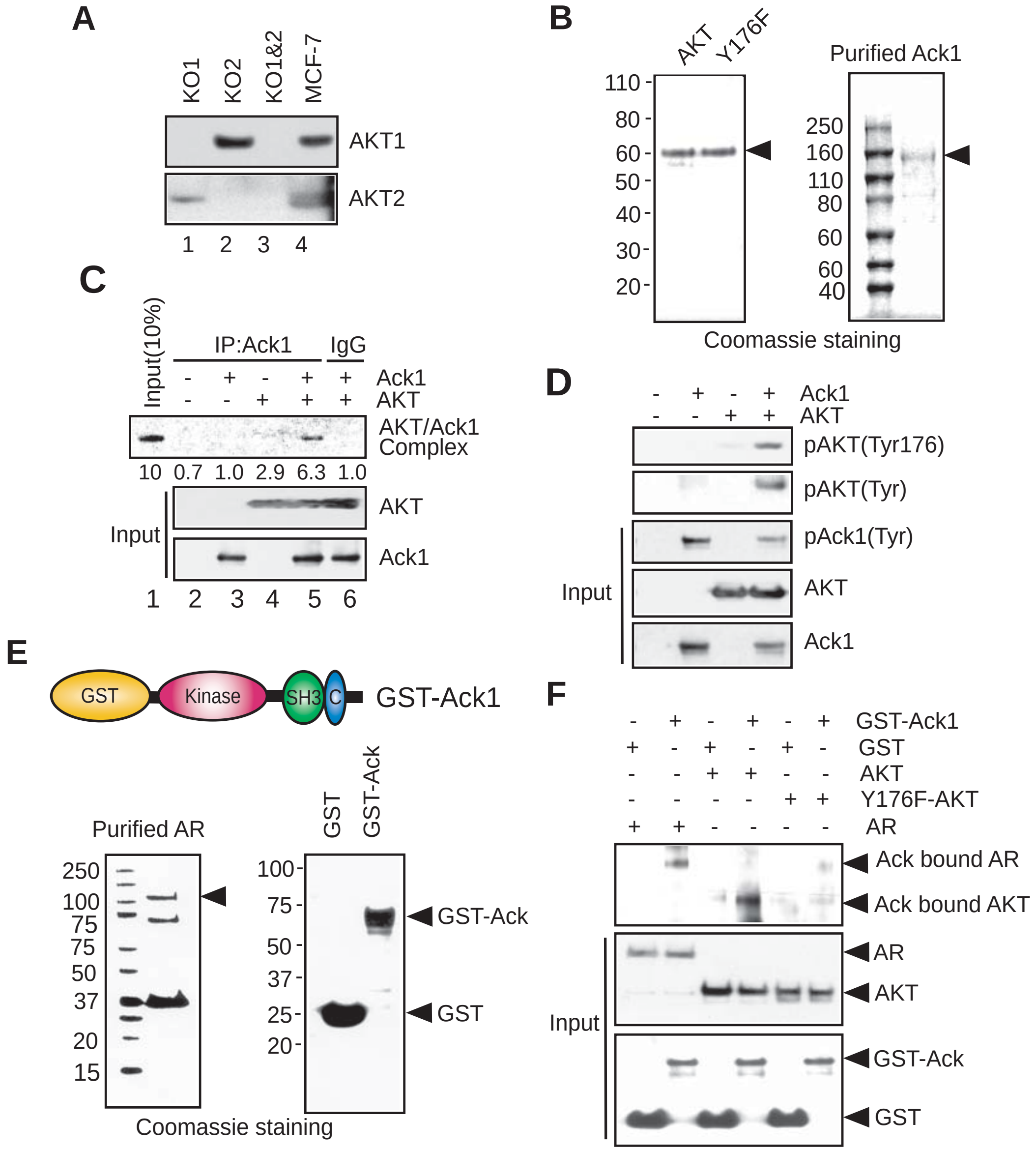

Figure S1. AKT is Tyr-phosphorylated by Ack1 *in vitro*. (A) AKT MEF KO1, KO2 and KO1&2s lack respective AKT isoforms. Equal amounts of MEFs protein lysates were subjected to IB as indicated. MCF-7 cell lysate was used as control. (**B**) Purification of Ack1 and AKT. HA-tagged Ack1 and AKT were expressed in HEK293T cells, lysed and incubated with HA-beads. Followed by extensive washing, proteins were eluted using HA-peptide (2nM, 1 hour) and assessed by SDS-PAGE and Coomassie Brilliant Blue-R250(BioRad) staining. **(C)** In vitro binding assay. Equimolar amounts of purified Ack1 and AKT proteins were incubated for 30 min, complex was immunoprecipitated with Ack1 (lanes 2-5) or IgG (lane#6) antibodies followed by IB with anti-AKT antibodies (top panel). About 6.35% of total AKT was in complex with Ack1. **(D)** In vitro phosphorylation of purified AKT by Ack1. Equimolar amounts of purified Ack1 and AKT proteins were incubated in kinase buffer for 1 hour at 37^0C and reaction mix was subjected to IB with pTyr176-AKT (top panel), pTyr (2nd and 3rd panels), AKT (4th panel) and Ack1 (bottom panel) antibodies. (**E**) Schematic representation of GST-Ack1 construct. FLAG-tagged AR expressed in HEK293 cells and GST-tagged Ack1 was expressed in DH5 cells. Purified GST-Ack1 (right panel) and FLAG-AR (left panel) were assessed by SDS-PAGE followed by Coomassie staining. **(F)** *In vitro* binding assay. Equimolar amounts of purified HA-AKT or FLAG-AR proteins were incubated with GST-Ack1 bound to beads for overnight, beads were washed followed by IB with anti-FLAG/HA antibodies (top panel). Lower panels show IB with FLAG/HA (2nd panel) and GST (bottom panel) antibodies.
