# Supplementary material for: Ack1 Mediated AKT/PKB Tyrosine 176 Phosphorylation Regulates Its Activation
Source: PLoS One. 2010 Mar 19;5(3):e9646. doi: 10.1371/journal.pone.0009646 (PMC2841635; doi:10.1371/journal.pone.0009646)
Supplement: Figure S2 — Tyr176-phosphorylated AKT sample also contains Thr308 and Ser473 phosphorylated AKT. (A) Activated Ack1 (caAck) and HA-tagged AKT were coexpressed in HEK293T cells followed by IP with HA-beads. IP AKT was subjected to SDS-PAGE electrophoresis and the gel was stained Coomassie. A prominent band of ∼59 kDa corresponding to AKT is seen which was excised and subjected to mass spectrometry as described in methods section. The upper ∼113 kDa band corresponds to caAck1 that bound to AKT. (B) Purified AKT peptide preparation that lead to the identification of pTyr176-AKT was assessed for other phosphorylation events. A peptide was detected at 21.12 mins in the total ion chromatogram with mass-to-charge ratio 918.43, which represents an error of 1.0 ppm (C). (D) The tandem mass spectrum matched the sequence, FGLCKEGIKDGATMKpTFC indicating that Thr308 in AKT was phosphorylated; the detection of the phosphothreonine y3 is consistent with this localization. (E) Another peptide was detected at 23.72 mins in the total ion chromatogram with mass-to-charge ratio 944.93, which represents an error of 0.99 ppm (F). (G) The tandem mass spectrum matched the sequence, ERRPHFPQFpSYSASGTA indicating that Ser473 in AKT was phosphorylated; the detection of b8, b9, y7 and y8 is consistent with this localization. (0.08 MB PDF) [file pone.0009646.s002.pdf]

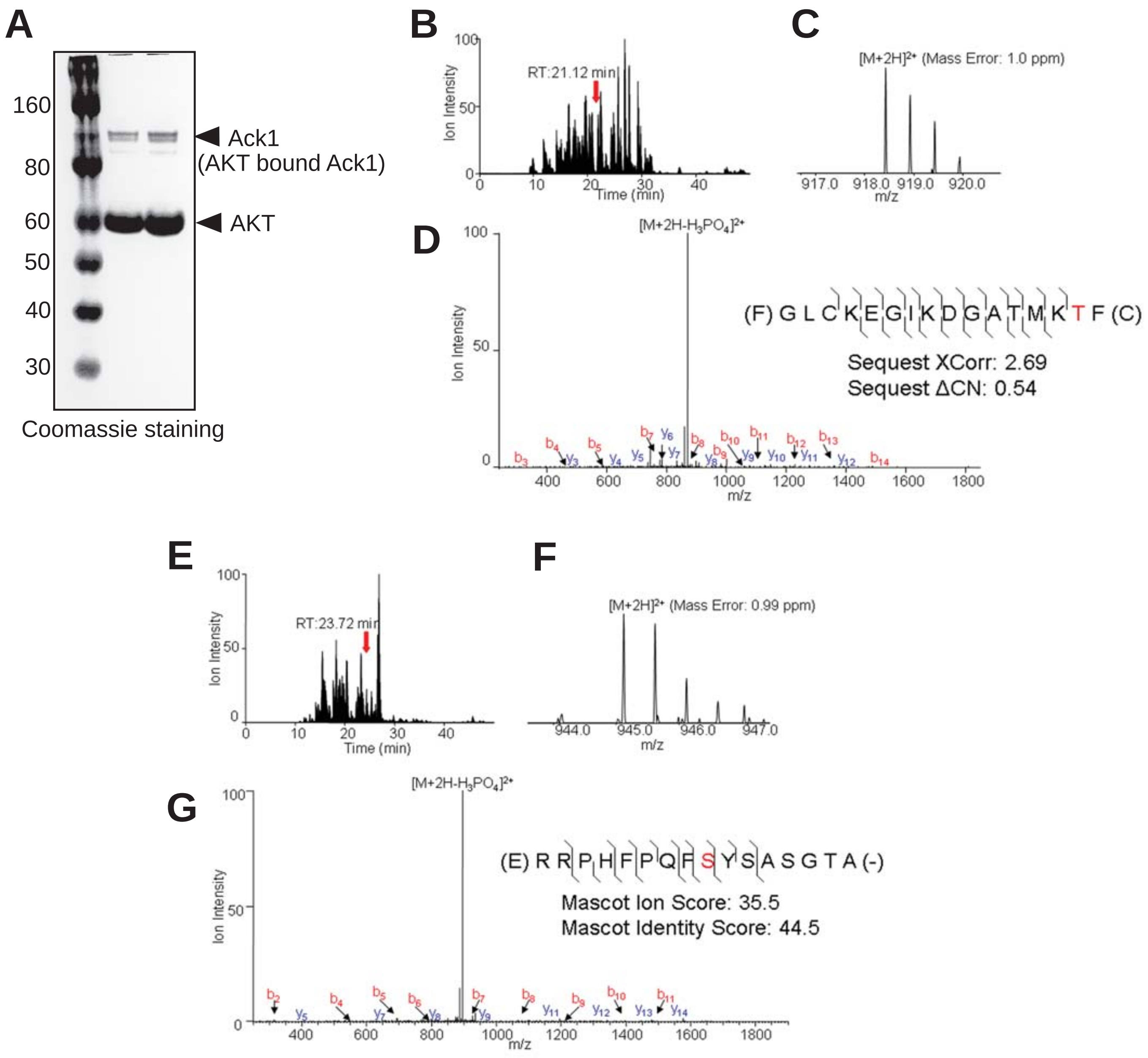

Figure S2. Tyr176-phosphorylated AKT sample also contains Thr308 and Ser473 phosphorylated AKT. (A) Activated Ack1 (caAck) and HA-tagged AKT were coexpressed in HEK293T cells followed by IP with HA-beads. IP AKT was subjected to SDS-PAGE electrophoresis and the gel was stained Coomassie. A prominent band of ~59 kDa corresponding to AKT is seen which was excised and subjected to mass spectrometry as described in methods section. The upper ~113 kDa band corresponds to caAck1 that bound to AKT. **(B)** Purified AKT peptide preparation that lead to the identification of pTyr176-AKT was assessed for other phosphorylation events. A peptide was detected at 21.12 mins in the total ion chromatogram with mass-to-charge ratio 918.43, which represents an error of 1.0 ppm (**C**). **(D)** The tandem mass spectrum matched the sequence, FGLCKEGIKDGATMK**pT**FC indicating that Thr308 in AKT was phosphorylated; the detection of the phosphothreonine y_3 is consistent with this localization. **(E)** Another peptide was detected at 23.72 mins in the total ion chromatogram with mass-to-charge ratio 944.93, which represents an error of 0.99 ppm (**F**). **(G)** The tandem mass spectrum matched the sequence, ERRPHFPQF**pS**YSASGTA indicating that Ser473 in AKT was phosphorylated; the detection of b_8, b_9, y_7 and y_8 is consistent with this localization.
