# Supplementary material for: Ack1 Mediated AKT/PKB Tyrosine 176 Phosphorylation Regulates Its Activation
Source: PLoS One. 2010 Mar 19;5(3):e9646. doi: 10.1371/journal.pone.0009646 (PMC2841635; doi:10.1371/journal.pone.0009646)
Supplement: Figure S3 — AKT Tyr176-phosphorylation affects the loop harboring Ser473. (A) Residues Tyr176 and Ser473 are located in regions with increased conformational flexibility. The backbone of AKT1 is color-traced according to crystallographic B-factors from blue (20 Angstrom, less flexible) to red (76 Angstrom, highly flexible). (B) B-factor plot of all C-alpha atoms. The average main chain B-factor is 36 Angstrom (dashed horizontal line). (C) AKT Tyr176-phosphorylation induces substantial conformational changes of residues in its vicinity. Electrostatic interactions could be established with Arg174 and/or Lys214 while electrostatic repulsion and/or steric hindrance (due to the bulky phosphate group) may affect Glu169 and Tyr215. This could lead to a shift of the beta-strand flanking the c-terminal portion of the loop harboring Ser473, in turn causing structural alterations of this residue. (0.07 MB PDF) [file pone.0009646.s003.pdf]

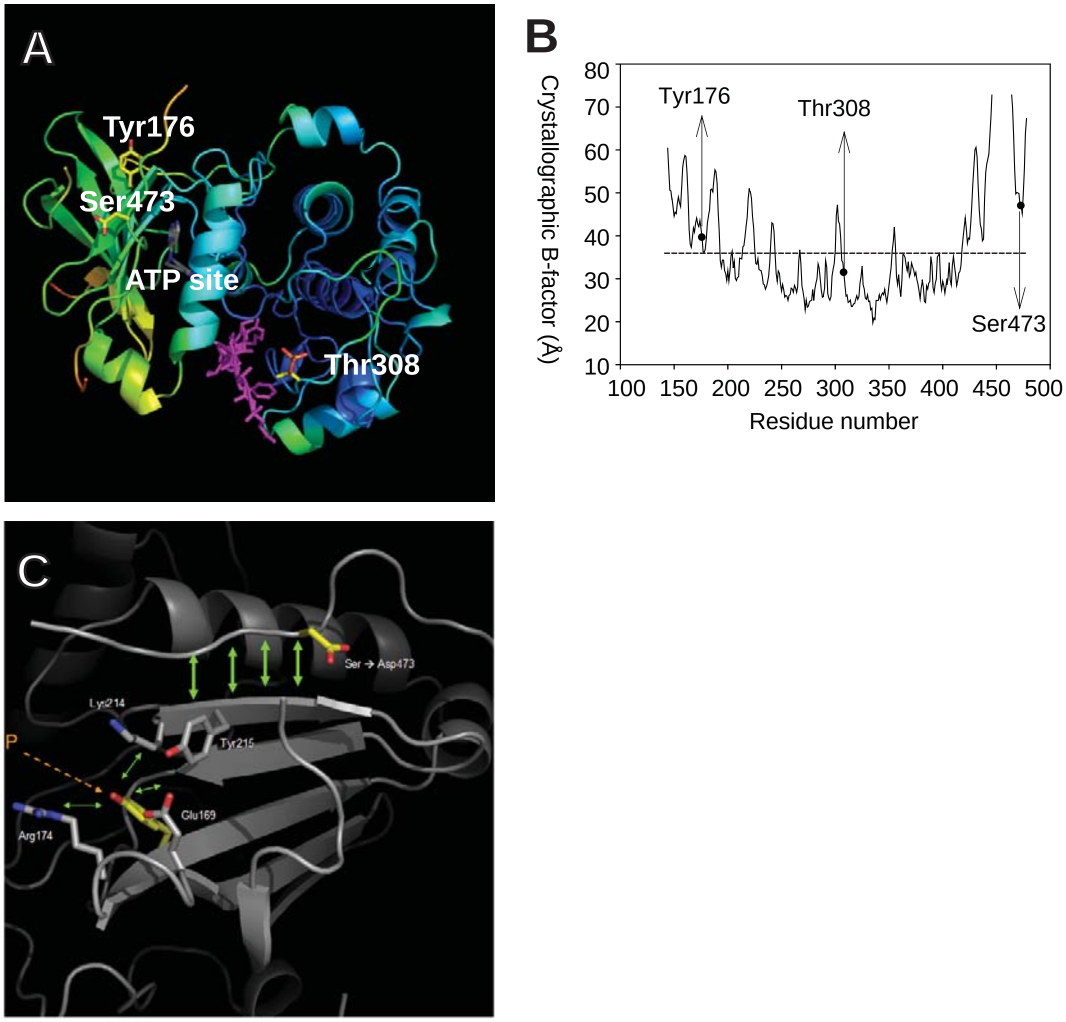


**Fig. S3. AKT Tyr176-phosphorylation affects the loop harboring Ser473.** (*A*) Residues Tyr176 and Ser473 are located in regions with increased conformational flexibility. The backbone of AKT1 is color-traced according to crystallographic B-factors from blue (20Å, less flexible) to red (76Å, highly flexible). (*B*) B-factor plot of all C-alpha atoms. The average main chain B-factor is 36Å (dashed horizontal line). (*C*) AKT Tyr176-phosphorylation induces substantial conformational changes of residues in its vicinity. Electrostatic interactions could be established with Arg174 and/or Lys214 while electrostatic repulsion and/or steric hindrance (due to the bulky phosphate group) may affect Glu169 and Tyr215. This could lead to a shift of the β-strand flanking the c-terminal portion of the loop harboring Ser473, in turn causing structural alterations of this residue.
