# Supplementary material for: Ack1 Mediated AKT/PKB Tyrosine 176 Phosphorylation Regulates Its Activation
Source: PLoS One. 2010 Mar 19;5(3):e9646. doi: 10.1371/journal.pone.0009646 (PMC2841635; doi:10.1371/journal.pone.0009646)
Supplement: Figure S4 — Kinase domain of Ack1 interacts with AKT PH domain/Tyr176 in kinase domain. (A) Schematic representation of wild type AKT, Y176F point mutant and deletion constructs. Site-directed mutagenesis of AKT was performed to generate the tyrosine to phenylalanine, Y176F, point mutant. PH, Pleckstrin homology domain; Kinase, Kinase domain and CT, Carboxy Terminal regulatory region. Schematic representation of Ack1 and deletion constructs. SAM, Sterile alpha motif; Kinase, kinase domain; SH3, Src homology domain 3; C, Cdc42 Rac interactive binding domain. (B) Flow cytometry of AKT 1&2KOMEFs, expressing HA-AKT and/or HA-Y176F. Top left panel indicates mock transfected cells stained with AKT-Ser473 antibody conjugated to Alexa 647 (untreated: 0.1%). Bottom left panel shows percentage of cells with AKT Ser473-phosphorylation upon EGF stimulation (15.2%). Right top and bottom panels show percentage of cells expressing HA-AKT (23%) or HA-Y176F (31%), respectively, in cells stained with anti-HA antibody conjugated to Alexa 488. (C) MEF1&2KO cells were co-transfected with HA-tagged AKT deletions and caAck1. The lysates were IP using HA antibodies followed by IB with pTyr antibodies (top panel). Lower panel show IP using HA antibodies followed by IB with AKT antibodies. Bottom panel show IB of the lysate with Ack1 antibodies. (D) HEK293 cells were co-transfected with HA-tagged AKT deletions and myc-tagged caAck. The lysates were IP using Myc antibodies followed by IB with HA antibodies (top panel). Lower panels are as described above. (E) MEF1&2KO cells were transfected with myc-tagged Ack1 deletions and HA-tagged AKT. The lysates were IP using Myc antibodies followed by IB with AKT antibodies (top panel). Lower panels show IB with Myc and AKT antibodies. (0.08 MB PDF) [file pone.0009646.s004.pdf]

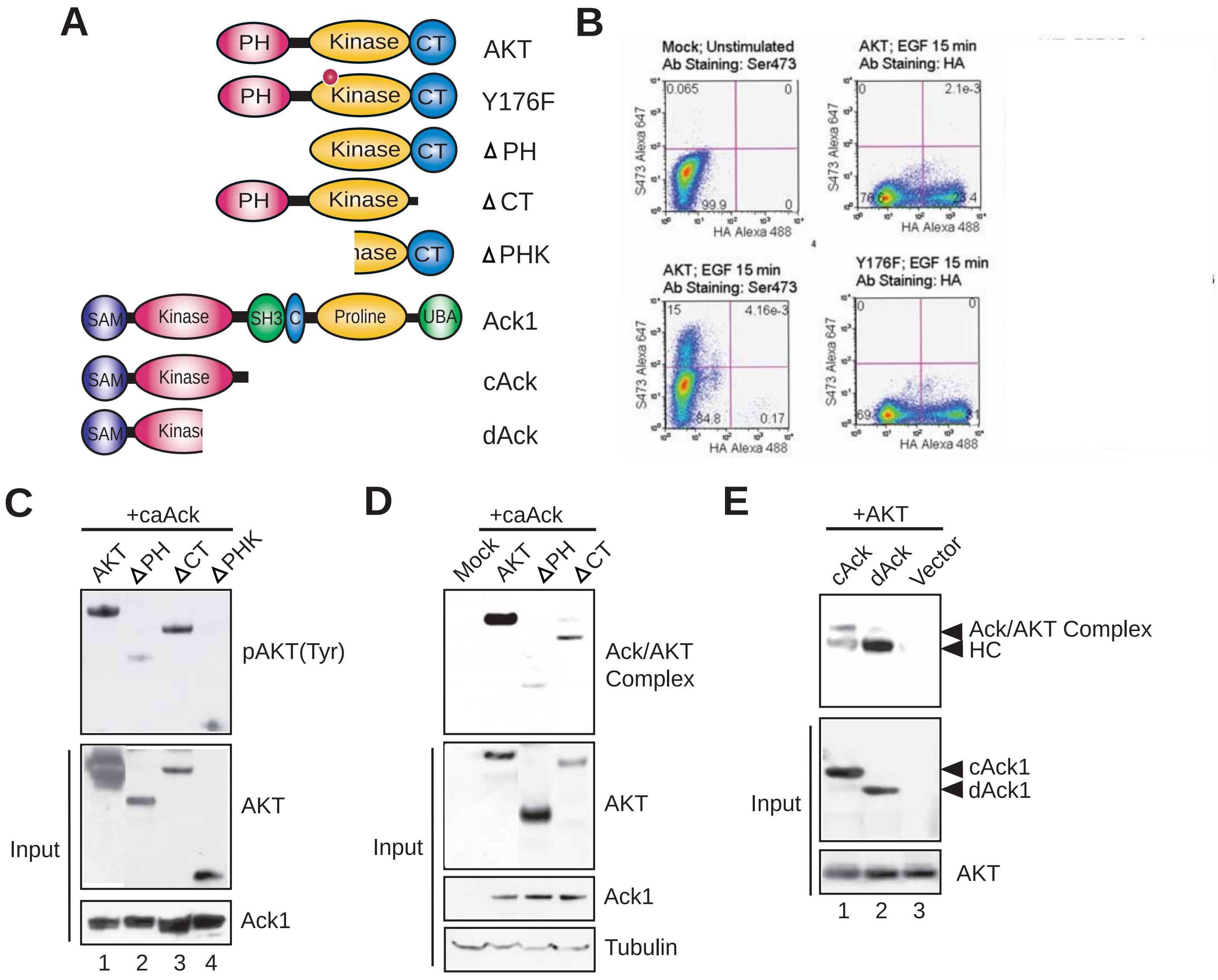

Figure S4. Kinase domain of Ack1 interacts with AKT PH domain/Tyr176 in kinase domain. (A) Schematic representation of wild type AKT, Y176F point mutant and deletion constructs. Site-directed mutagenesis of AKT was performed to generate the tyrosine to phenylalanine, Y176F, point mutant. PH, Pleckstrin homology domain; Kinase, Kinase domain and CT, Carboxy Terminal regulatory region. Schematic representation of Ack1 and deletion constructs. SAM, Sterile alpha motif; Kinase, kinase domain; SH3, Src homology domain 3; C, Cdc42 Rac interactive binding domain. **(B)** Flow cytometry of AKT 1&2KOMEFs, expressing HA-AKT and/or HA-Y176F. Top left panel indicates mock transfected cells stained with AKT-Ser473 antibody conjugated to Alexa 647 (untreated: 0.1%). Bottom left panel shows percentage of cells with AKT Ser473-phosphorylation upon EGF stimulation (15.2%). Right top and bottom panels show percentage of cells expressing HA-AKT (23%) or HA-Y176F (31%), respectively, in cells stained with anti-HA antibody conjugated to Alexa 488. (**C**) MEF1&2KO cells were co-transfected with HA-tagged AKT deletions and caAck1. The lysates were IP using HA antibodies followed by IB with pTyr antibodies (top panel). Lower panel show IP using HA antibodies followed by IB with AKT antibodies. Bottom panel show IB of the lysate with Ack1 antibodies. (**D**) HEK293 cells were co-transfected with HA-tagged AKT deletions and myc-tagged caAck. The lysates were IP using Myc antibodies followed by IB with HA antibodies (top panel). Lower panels are as described above. (**E**) MEF1&2KO cells were transfected with myc-tagged Ack1 deletions and HA-tagged AKT. The lysates were IP using Myc antibodies followed by IB with AKT antibodies (top panel). Lower panels show IB with Myc and AKT antibodies.
