# Supplementary material for: Ack1 Mediated AKT/PKB Tyrosine 176 Phosphorylation Regulates Its Activation
Source: PLoS One. 2010 Mar 19;5(3):e9646. doi: 10.1371/journal.pone.0009646 (PMC2841635; doi:10.1371/journal.pone.0009646)
Supplement: Figure S5 — Somatic autoactivation of Ack1. (A) Schematic representation of Ack1 and various point mutants identified in the COSMIC database. Site-directed mutagenesis of Ack1 was performed to generate four HA-tagged point mutants. SAM, Sterile alpha motif; Kinase, kinase domain; SH3, Src homology domain 3; C, Cdc42 Rac interactive binding domain; Proline, Proline rich domain; UBA, Ubiquitin binding domain. (B) E346K mutation results in Ack1 autoactivation leading to AKT activation. MEF1&2KO cells were transfected with Ack1 mutants and the lysates were IP using anti-HA antibodies followed by IB with pTyr antibodies (top panel). Lower panels show IB with indicated antibodies. (C) E346K mutant Ack1 interacts with and Tyr-phosphorylates AKT. 293T cells were co-transfected with HA-tagged Ack1 point mutants. Equal amounts of protein lysates were subjected to IP using HA antibodies. IB with AKT antibodies revealed formation of activated Ack1(E346K)/endogenous AKT complex (top panel). (D) HEK293T cells were transfected with HA-tagged E346K, caAck or kdAck (K158R) mutants. Lysates were subjected to IP using anti-HA (top panel) antibodies followed by IB with pTyr284-Ack1 antibodies. Lower panels show IB with indicated antibodies. (E) E346K or caAck mediated AKT Tyr-phosphorylation leads to AKT kinase activation. HEK293T cells were co-transfected with E346K or myc-tagged caAck and AKT or Y176F mutant. Lysates were subjected to IP using anti-myc (top panel) and anti-Ack1 (second panel) antibodies followed by IB with pTyr antibodies. The same lysates were processed for kinase assay shown in S6F. (F) Ack1 autoactivation leads to AKT kinase activation. As described in S6E, lysates were IP with HA-antibodies, followed by AKT kinase assay. Low levels of Ack1 kinase activity in vector transfected cells was treated as zero and increased kinase activity (in percentage) over the vector expressing cells is shown. (0.05 MB PDF) [file pone.0009646.s005.pdf]

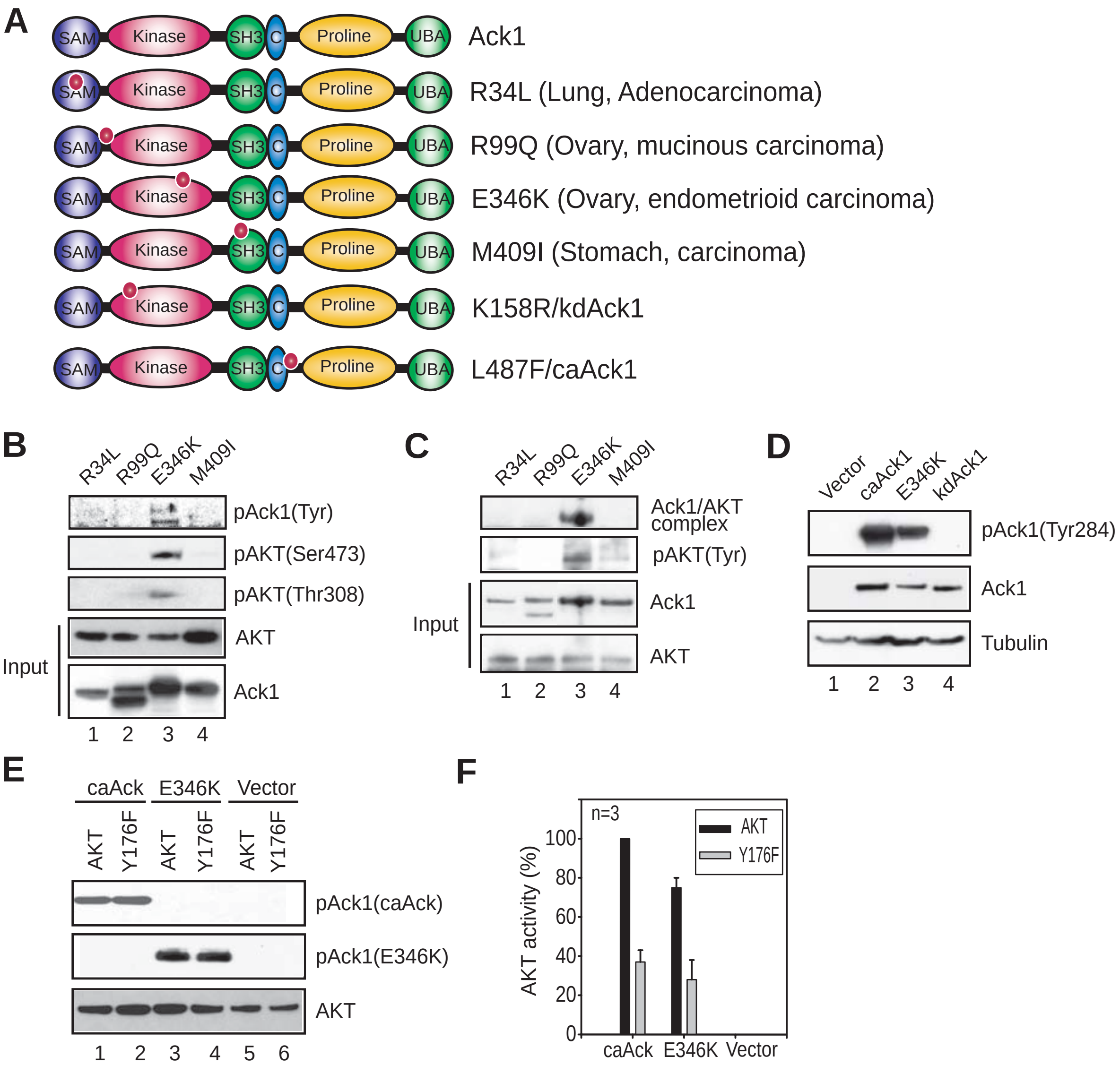


**Figure S5. Somatic autoactivation of Ack1. (A)** Schematic representation of Ack1 and various point mutants identified in the COSMIC database. Site-directed mutagenesis of Ack1 was performed to generate four HA-tagged point mutants. SAM, Sterile alpha motif; Kinase, kinase domain; SH3, Src homology domain 3; C, Cdc42 Rac interactive binding domain; Proline, Proline rich domain; UBA, Ubiquitin binding domain. **(B)** E346K mutation results in Ack1 autoactivation leading to AKT activation. MEF1&2KO cells were transfected with Ack1 mutants and the lysates were IP using anti-HA antibodies followed by IB with pTyr antibodies (top panel). Lower panels show IB with indicated antibodies. **(C)** E346K mutant Ack1 interacts with and Tyr-phosphorylates AKT. 293T cells were co-transfected with HA-tagged Ack1 point mutants. Equal amounts of protein lysates were subjected to IP using HA antibodies. IB with AKT antibodies revealed formation of activated Ack1(E346K)/endogenous AKT complex (top panel). **(D)** HEK293T cells were transfected with HA-tagged E346K, caAck or kdAck (K158R) mutants. Lysates were subjected to IP using anti-HA (top panel) antibodies followed by IB with pTyr284-Ack1 antibodies. Lower panels show IB with indicated antibodies. **(E)** E346K or caAck mediated AKT Tyr-phosphorylation leads to AKT kinase activation. HEK293T cells were co-transfected with E346K or myc-tagged caAck and AKT or Y176F mutant. Lysates were subjected to IP using anti-myc (top panel) and anti-Ack1 (second panel) antibodies followed by IB with pTyr antibodies. The same lysates were processed for kinase assay shown in S6F. **(F)** Ack1 autoactivation leads to AKT kinase activation. As described in S6E, lysates were IP with HA-antibodies, followed by AKT kinase assay. Low levels of Ack1 kinase activity in vector transfected cells was treated as zero and increased kinase activity (in percentage) over the vector expressing cells is shown.
