# Supplementary material for: Ack1 Mediated AKT/PKB Tyrosine 176 Phosphorylation Regulates Its Activation
Source: PLoS One. 2010 Mar 19;5(3):e9646. doi: 10.1371/journal.pone.0009646 (PMC2841635; doi:10.1371/journal.pone.0009646)
Supplement: Figure S6 — Generation and validation of pTyr176-AKT phospho-antibodies. (A) EGF and heregulin treatment results in AKT Tyr176-phosphorylation. RWPE, normal prostate epithelial cells were treated with EGF (10 ng/ml,10 mins) and heregulin (10 ng/ml, 35 mins) ligand, equal amounts of protein lysates were subjected to immunoblotting as indicated. pTyr176-antibodies specifically recognizes endogenous Tyr-phosphorylated AKT following treatment with ligands. (B) Ack1 activation lead to AKT Tyr176-phosphorylation. 293T cells were co-transfected with myc-tagged caAck or kdAck and AKT or Y176F mutant. Equal amounts of protein lysates were subjected to immunoblotting with pTyr176-AKT antibodies. The pTyr176-antibodies recognize only the pTyrAKT (lane 2), but not the Y176F point mutant (lane 4). (C-J) Tyr176-phosphorylated AKT localizes at plasma membrane. NIH3T3 cells were co-transfected with EGFP-E346K mutant of Ack1 and dsRed2-N1-AKT (D-F) or dsRed2-N1-Y176F-AKT (G-J) DNAs overnight. Cells were serum starved, fixed and visualized by fluorescence microscopy. AKT but not Y176F mutant was localized to the plasma membrane in activated Ack1(E346K) expressing cells. (0.07 MB PDF) [file pone.0009646.s006.pdf]

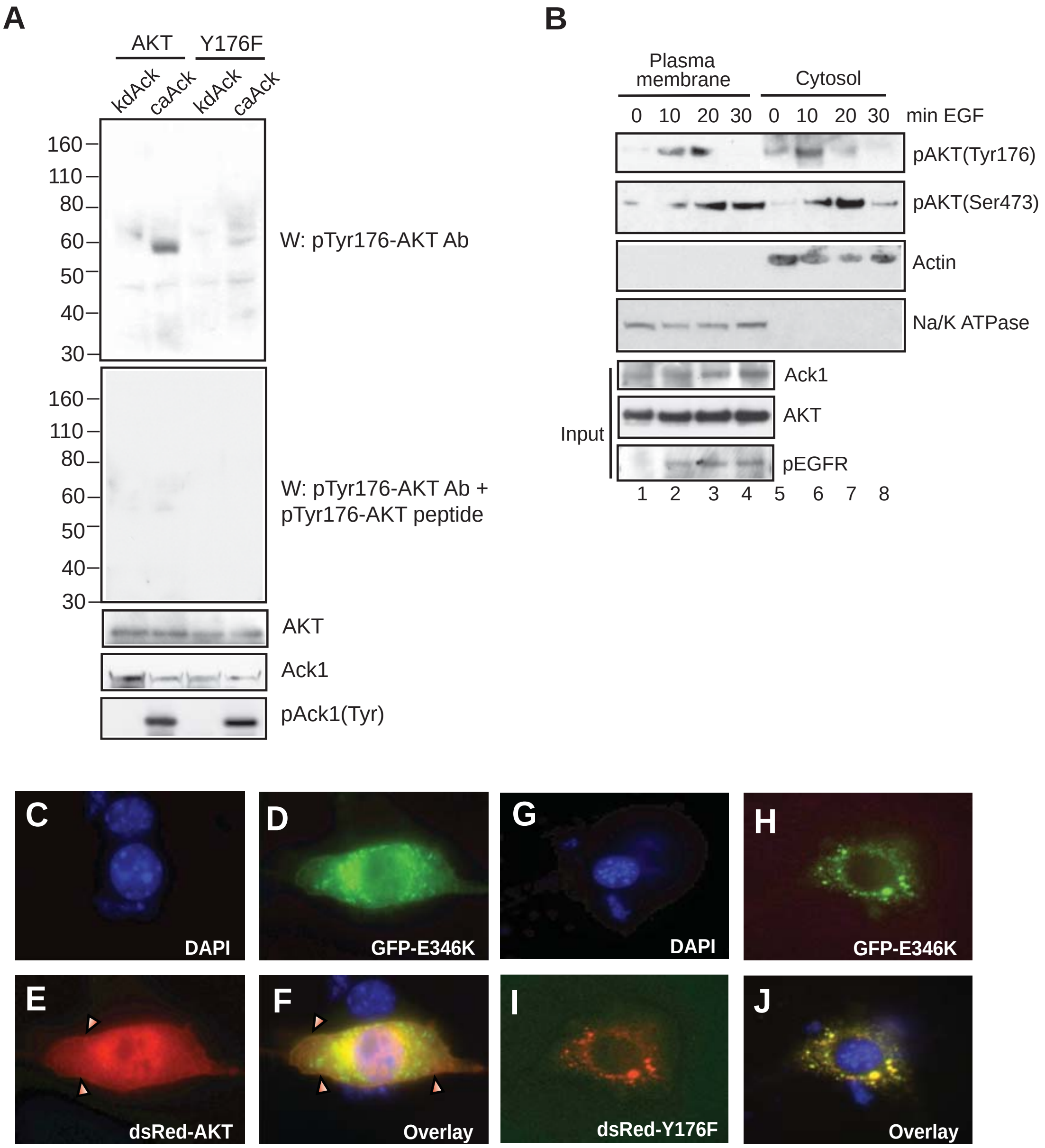

Fig. S6. Validation of pTyr176-AKT phospho-antibodies and pTyr176-AKT localizationto plasma membrane (*A*) Ack1 activation lead to AKT Tyr176-phosphorylation. 293T cells were co-transfected with myc-tagged caAck or kdAck and AKT or Y176F mutant. Equal amounts of whole protein lysates were subjected to immunoblotting with pTyr176-AKT antibodies (top panel). The pTyr176-antibodies recognize only the pTyrAKT (lane 2), but not the Y176F point mutant (lane 4). Similarly, equal amounts of whole protein lysates were subjected to immunoblotting with pTyr176-AKT antibodies that were preincubated with AKT phosphopeptide for 30 min (second panel). The pTyr176-antibodies blocked by AKT phosphopeptide failed to recognize pTyr176-AKT (lane 2). (*B*) RWPE cells were treated with EGF (10 ng/ml) for various time intervals and cell lysates were fractionated into plasma membrane and cytosolic fractions. Equal amounts of protein from these two fractions were subjected to immunoblotting with indicated antibodies. Tyr176-phosphorylated-AKT accumulates at the membrane upon 10 min of EGF addition. (*C-J*) Tyr176-phosphorylated AKT localizes at plasma membrane. NIH3T3 cells were co-transfected with EGFP-E346K mutant of Ack1 and dsRed2-N1-AKT (*C-F*) or dsRed2-N1-Y176F-AKT (*G-J*) DNAs overnight. Cells were serum starved, fixed and visualized by fluorescence microscopy. AKT but not Y176F mutant was localized to the plasma membrane in activated Ack1(E346K) expressing cells.
