# Supplementary material for: Ack1 Mediated AKT/PKB Tyrosine 176 Phosphorylation Regulates Its Activation
Source: PLoS One. 2010 Mar 19;5(3):e9646. doi: 10.1371/journal.pone.0009646 (PMC2841635; doi:10.1371/journal.pone.0009646)
Supplement: Figure S7 — Tyr176-phosphorylation of mutant AKT (R25C) that inefficiently binds phosphatidyl-inositol 3,4, 5-triphosphate. (A) MEF1&2KO cells were transfected with activated Ack and AKT followed by LY294002 (10 µM) for 1 h. Cell lysates were fractionated and subjected to immunoblotting with indicated antibodies. AKT Ser473 phosphorylation in membrane fraction was unaffected by LY294002 treatment suggesting Ack1 mediated AKT activation is not dependent upon PI3K activity. (B) Schematic representation of wild type AKT and R25C point mutant constructs. Site-directed mutagenesis of AKT was performed to generate the R25C point mutant. PH, Pleckstrin homology domain; Kinase, Kinase domain and CT, Carboxy Terminal regulatory region. (C) MEF1&2 KO cells were transfected with empty vector or caAck and HA-tagged AKT or R25C mutant DNAs. Serum starved (18 h) cells were treated with EGF (10 ng/ml, 15 mins). The lysates were subjected to immunoprecipitation with anti-HA (top panel) or anti-Ack1 (second panel) antibodies followed by immunoblotting with pTyr antibodies. (D) MEF1&2 KO cells were transfected with empty vector or caAck and HA-tagged AKT or R25C mutant DNAs. Serum starved (18 h) cells were treated with EGF (10 ng/ml, 15 min). Cell lysates were fractionated and subjected to immunoblotting. (0.05 MB PDF) [file pone.0009646.s007.pdf]

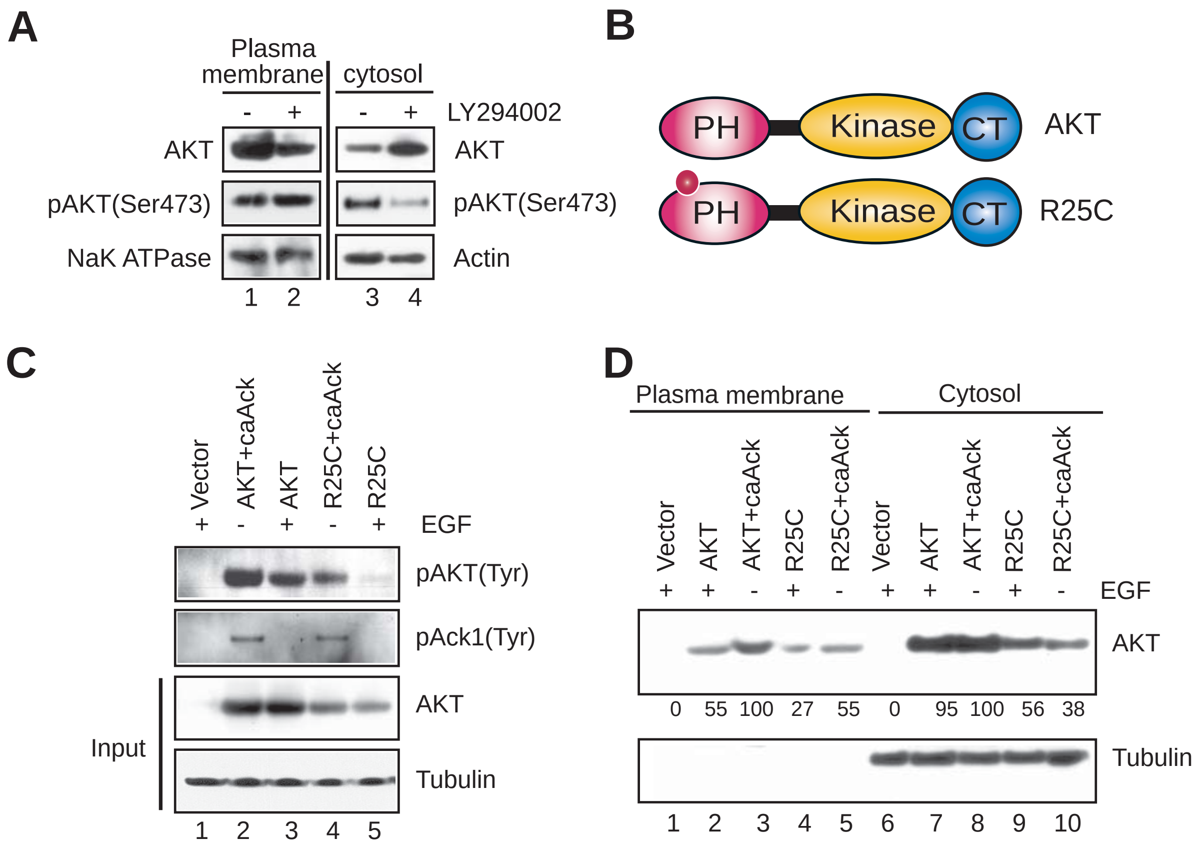

Fig. S7. Tyr176-phosphorylation of mutant AKT (R25C) that inefficiently binds phosphatidyl-inositol 3,4, 5-triphosphate. (*A*) MEF1&2KO cells were transfected with activated Ack and AKT followed by LY294002 (10µM) for 1h. Cell lysates were fractionated and subjected to immunoblotting with indicated antibodies. AKT Ser473 phosphorylation in membrane fraction was unaffected by LY294002 treatment suggesting Ack1 mediated AKT activation is not dependent upon PI3K activity. (*B*) Schematic representation of wild type AKT and R25C point mutant. Site-directed mutagenesis of AKT was performed to generate the arginine to cystine, R25C, point mutant. PH, Pleckstrin homology domain; Kinase, Kinase domain and CT, Carboxy Terminal regulatory region. (*C*) MEF1&2 KO cells were transfected with empty vector or caAck and HA-tagged AKT or R25C mutant DNAs. Serum starved (18 h) cells were treated with EGF (10ng/ml, 15 mins). The lysates were subjected to immunoprecipitation with anti-HA (top panel) or anti-Ack1 (second panel) antibodies followed by immunoblotting with pTyr antibodies. (*D*) MEF1&2 KO cells were transfected with empty vector or caAck and HA-tagged AKT or R25C mutant DNAs. Serum starved (18 h) cells were treated with EGF (10ng/ml, 15 min). Cell lysates were fractionated and subjected to immunoblotting.
