# Supplementary material for: Ack1 Mediated AKT/PKB Tyrosine 176 Phosphorylation Regulates Its Activation
Source: PLoS One. 2010 Mar 19;5(3):e9646. doi: 10.1371/journal.pone.0009646 (PMC2841635; doi:10.1371/journal.pone.0009646)
Supplement: Figure S8 — Tyr-phosphorylated AKT binds to phosphatidic acid. Protein-phospholipid overlay assay was performed using nitrocellulose membranes spotted with 100 pmol of different phospholipids. (A-C, F,G) Cells transfected with vector or activated Ack1 and AKT or Y176F were lysed and immunoprecipitated with pTyr-beads followed by elution with phenylphosphate. The eluted Tyr-phosphorylated proteins were incubated with phospholipid blots overnight at 4°C. Blots were extensively washed and bound proteins were detected with (A, B and F) pTyr176-AKT and (C and G) AKT antibodies. (D and E) Cells expressing HA-tagged AKT (D) and Y176F mutant (E) were lysed and immunoprecipitated with HA-beads followed by elution with HA peptide. The eluate was incubated with phospholipid blot and bound protein was detected with AKT antibodies. The pTyr176-AKT bound to phosphatidic acid, in contrast, AKT protein primarily binds to phosphatidyl-inositol 3,4,5-triphosphate (PIP3). (H) HA-peptide and phenylphosphate eluates were immunoblotted with antibodies shown to confirm presence of desired proteins. (0.03 MB PDF) [file pone.0009646.s008.pdf]

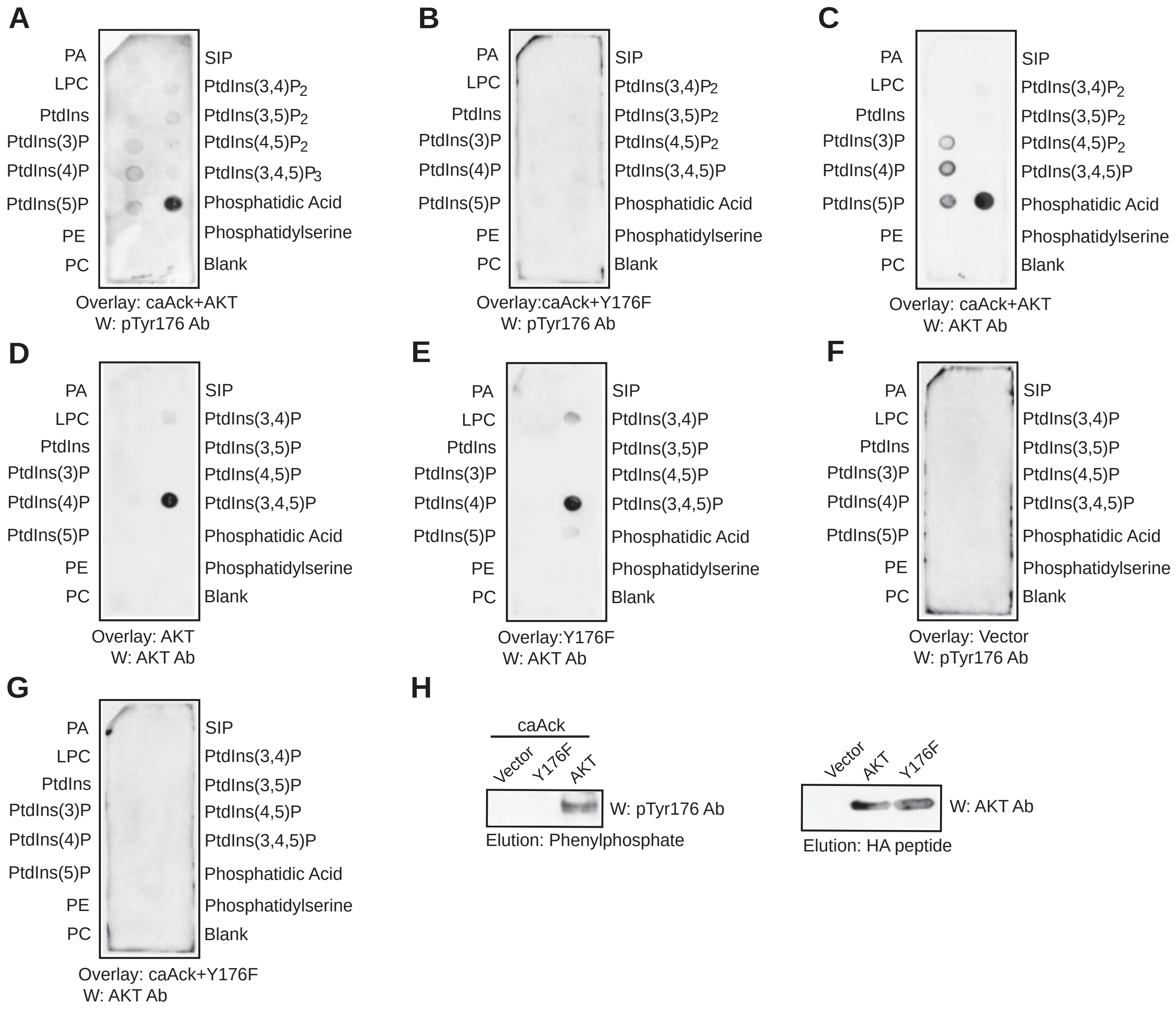

Figure S8. Tyr-phosphorylated AKT binds to phosphatidic acid. Protein-phospholipid overlay assay was performed using nitrocellulose membranes spotted with 100 pmol of different phospholipids. **(A-C, F-G)** Cells transfected with vector or activated Ack1 and AKT or Y176F were lysed and immunoprecipitated with pTyr-beads followed by elution with phenylphosphate. The eluted Tyr-phosphorylated proteins were incubated with phospholipid blots overnight at 4°C. Blots were extensively washed and bound proteins were detected with **(A, B and F)** pTyr176-AKT and **(C and G)** AKT antibodies**. (D and E)** Cells expressing HA-tagged **(D)** AKT and **(E)** Y176F mutant AKT were lysed and immunoprecipitated with HA-beads followed by elution with HA peptide. The eluate was incubated with phospholipid blots and bound proteins were detected with AKT antibodies**.** The pTyr176-AKT bound to phosphatidic acid, in contrast, AKT and Y176F mutant proteins bound primarily to phosphatidyl-inositol 3,4,5-triphosphate. **(H)** HA peptide and phenylphosphate eluate was immunoblotted with antibodies shown to confirm presence of desired proteins.
