# Supplementary material for: Ack1 Mediated AKT/PKB Tyrosine 176 Phosphorylation Regulates Its Activation
Source: PLoS One. 2010 Mar 19;5(3):e9646. doi: 10.1371/journal.pone.0009646 (PMC2841635; doi:10.1371/journal.pone.0009646)
Supplement: Figure S9 — Tyr176 phosphorylated AKT is enriched in the nucleus. (A) MCF-7 cells were serum starved (24 h) and treated with heregulin (30 ng/ml) for indicated times. Cell lysates were fractionated into nuclear and cytoplasmic fractions. Equal amounts of protein from these two fractions were subjected to immunoblotting with indicated Abs. Activated Ack1 mediated Tyr176 phosphorylated AKT is enriched in the nucleus 45 mins after heregulin treatment. The mobility of pTyr176-AKT is affected due to difference in the salt concentrations of nuclear (300 mM NaCl) and cytoplasmic fractions (10 mM KCl) (top panel). (B) MEF1&2KO cells were transfected with HA-tagged myr-AKT or myr-Y176F, equal amounts of protein lysates were subjected to immunoblotting as indicated. The myristoylated-AKT exhibits high levels of AKT activation, as seen by Thr308-phosphorylation. (0.03 MB PDF) [file pone.0009646.s009.pdf]

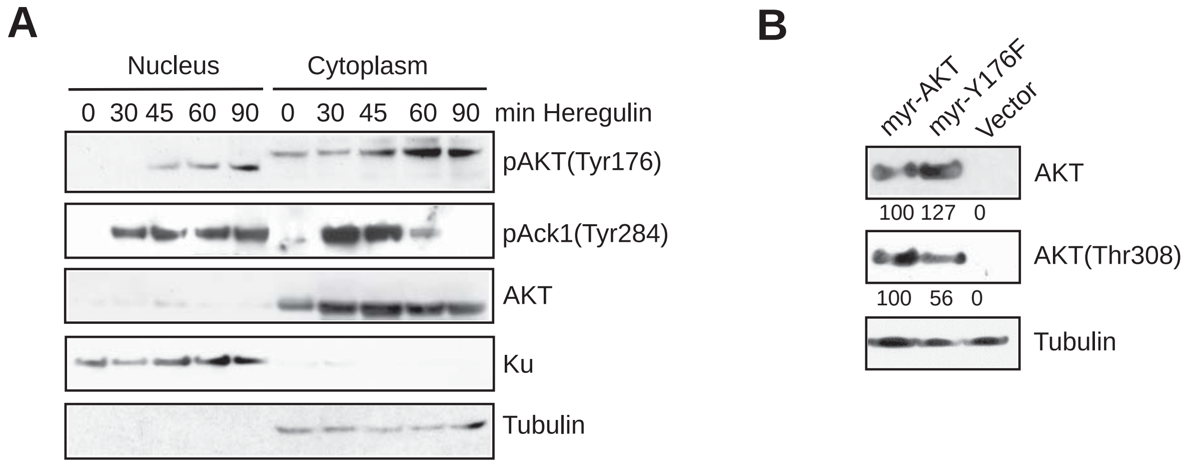

Fig. S9. (*A*) **Tyr176 phosphorylated AKT is enriched in the nucleus.** MCF-7 cells were serum starved (24 h) and treated with heregulin (30 ng/ml) for indicated times. Cell lysates were fractionated into nuclear and cytoplasmic fractions. Equal amounts of protein from these two fractions were subjected to immunoblotting with indicated Abs. Activated Ack1 mediated Tyr176 phosphorylated AKT is enriched in the nucleus 45 mins after heregulin treatment. The mobility of pTyr176-AKT is affected due to difference in the salt concentrations of nuclear (300mM NaCl) and cytoplasmic fractions (10mM KCl) (top panel). (*B*) MEF1&2KO cells were transfected with HA-tagged myr-AKT or myr-Y176F, equal amounts of protein lysates were subjected to immunoblotting as indicated. The myristoylated-AKT exhibits high levels of AKT activation, as seen by Thr308-phosphorylation.
