# Supplementary material for: Ack1 Mediated AKT/PKB Tyrosine 176 Phosphorylation Regulates Its Activation
Source: PLoS One. 2010 Mar 19;5(3):e9646. doi: 10.1371/journal.pone.0009646 (PMC2841635; doi:10.1371/journal.pone.0009646)
Supplement: Figure S10 — Staining of tumor samples with Tyr284-phosphorylated-Ack1 and Tyr176-phosphorylated-AKT antibodies. Representations of Tyr284-phosphorylated-Ack1 (A) and Tyr176-phosphorylated-AKT (B) staining of IDC, which show intense staining in nuclei and membrane. (C) Expression levels between Tyr284-phosphorylated-Ack1 and Tyr176-phosphorylated-AKT expression were significantly correlated in breast tumors (Spearman rank correlation coefficient rho = 0.43, p<0.0001). (D–G) Breast samples stained with Ack1 and pAck1(Tyr284) antibodies. Basal levels of Ack1 expression were seen in both normal and tumor samples (D, E), however, significant increase in pAck1(Tyr284) staining was seen in tumor samples as contrast to normal breast sample (compare F and G). (0.10 MB PDF) [file pone.0009646.s010.pdf]

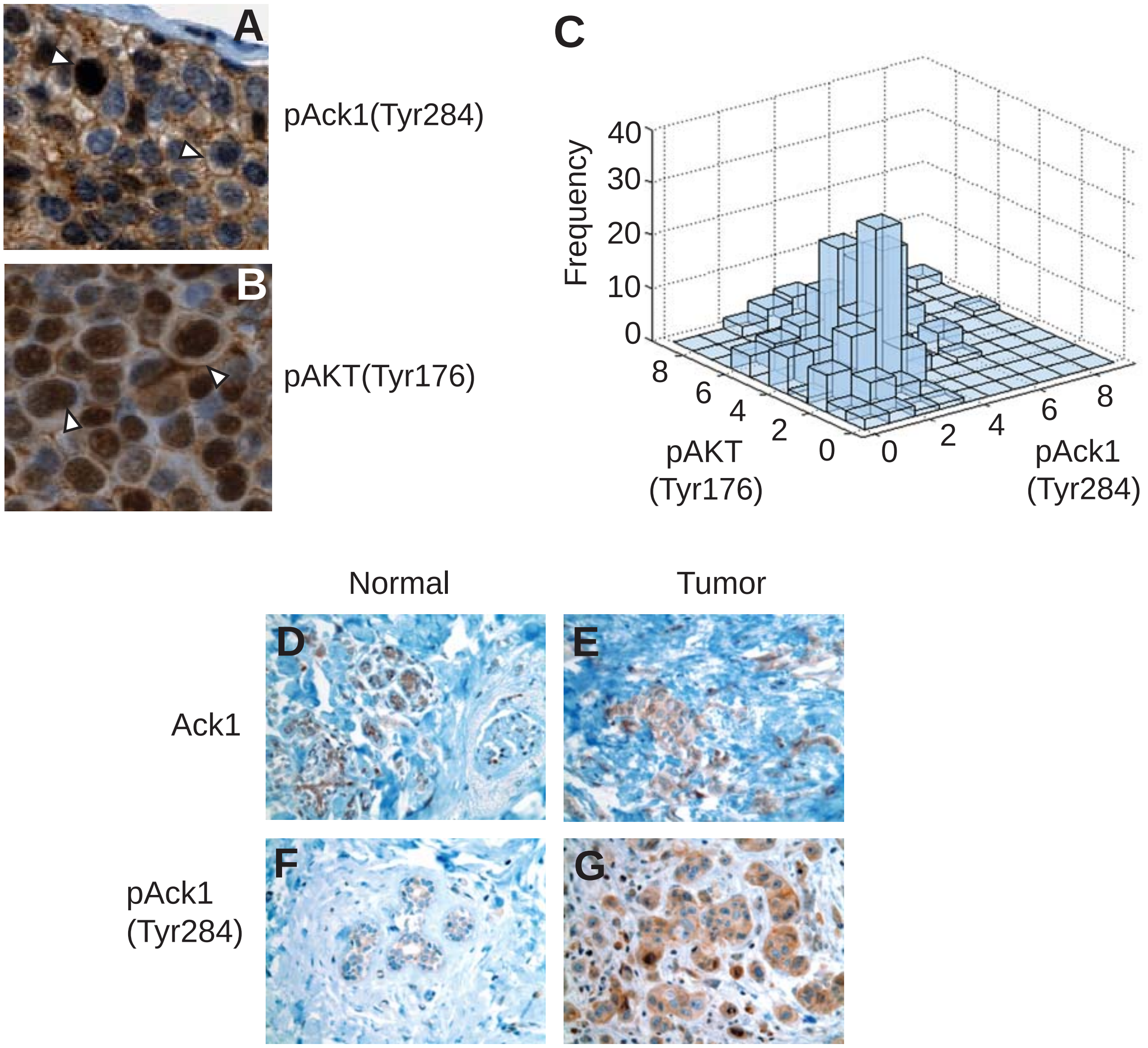

Figure S10. Staining of tumor samples with Tyr284-phosphorylated-Ack1 and Tyr176-phosphorylated-AKT antibodies. Representations of Tyr284-phosphorylated-Ack1 (**A**) and Tyr176-phosphorylated-AKT (**B**) staining of IDC, which show intense staining in nuclei and membrane. (**C**) Expression levels between Tyr284-phosphorylated-Ack1 and Tyr176-phosphorylated-AKT expression were significantly correlated in breast tumors (Spearman rank correlation coefficient $\rho = 0.43$, $p < 0.0001$). **(D-G)** Breast samples stained with Ack1 and pAck1(Tyr284) antibodies. Basal levels of Ack1 expression were seen in both normal and tumor samples **(D, E)**, however, significant increase in pAck1(Tyr284) staining was seen in tumor samples as contrast to normal breast sample (compare **F** and **G**).
